# Regulation of RLR-Mediated Antiviral Responses of Human Dendritic Cells by mTOR

**DOI:** 10.3389/fimmu.2020.572960

**Published:** 2020-09-11

**Authors:** Tünde Fekete, Beatrix Ágics, Dóra Bencze, Krisztián Bene, Antónia Szántó, Tünde Tarr, Zoltán Veréb, Attila Bácsi, Kitti Pázmándi

**Affiliations:** ^1^Department of Immunology, Faculty of Medicine, University of Debrecen, Debrecen, Hungary; ^2^Doctoral School of Molecular Cell and Immune Biology, University of Debrecen, Debrecen, Hungary; ^3^Division of Clinical Immunology, Faculty of Medicine, University of Debrecen, Debrecen, Hungary; ^4^Department of Dermatology and Allergology, Regenerative Medicine and Cellular Pharmacology Laboratory, Faculty of Medicine, University of Szeged, Szeged, Hungary

**Keywords:** dendritic cell, mTOR, RLR signaling, antiviral response, T cell stimulation

## Abstract

To detect replicating viruses, dendritic cells (DCs) utilize cytoplasmic retinoic acid inducible gene-(RIG) I-like receptors (RLRs), which play an essential role in the subsequent activation of antiviral immune responses. In this study, we aimed to explore the role of the mammalian target of rapamycin (mTOR) in the regulation of RLR-triggered effector functions of human monocyte-derived DCs (moDCs) and plasmacytoid DCs (pDCs). Our results show that RLR stimulation increased the phosphorylation of the mTOR complex (mTORC) 1 and mTORC2 downstream targets p70S6 kinase and Akt, respectively, and this process was prevented by the mTORC1 inhibitor rapamycin as well as the dual mTORC1/C2 kinase inhibitor AZD8055 in both DC subtypes. Furthermore, inhibition of mTOR in moDCs impaired the RLR stimulation-triggered glycolytic switch, which was reflected by the inhibition of lactate production and downregulation of key glycolytic genes. Blockade of mTOR diminished the ability of RLR-stimulated moDCs and pDCs to secret type I interferons (IFNs) and pro-inflammatory cytokines, while it did not affect the phenotype of DCs. We also found that mTOR blockade decreased the phosphorylation of Tank-binding kinase 1 (TBK1), which mediates RLR-driven cytokine production. In addition, rapamycin abrogated the ability of both DC subtypes to promote the proliferation and differentiation of IFN-y and Granzyme B producing CD8 + T cells. Interestingly, AZD8055 was much weaker in its ability to decrease the T cell proliferation capacity of DCs and was unable to inhibit the DC-triggered production of IFN-y and Granyzme B by CD8 + T cells. Here we demonstrated for the first time that mTOR positively regulates the RLR-mediated antiviral activity of human DCs. Further, we show that only selective inhibition of mTORC1 but not dual mTORC1/C2 blockade suppresses effectively the T cell stimulatory capacity of DCs that should be considered in the development of new generation mTOR inhibitors and in the improvement of DC-based vaccines.

## Introduction

Dendritic cells (DCs) play an essential role in the initiation of efficient immune responses against viral infections. As sentinels of the immune system DCs express a diverse set of pattern recognition receptors (PRRs) including Toll-like receptors (TLRs) and retinoic acid inducible gene-(RIG) I-like receptors (RLRs), which makes them able to recognize and bind to a wide range of viral nucleic acids and other conserved molecular signatures (1, 2). Upon recognition of viral infection, DCs undergo profound phenotypic and functional changes, which endows them with the ability to successfully mount an antiviral state and the capability to migrate to secondary lymphoid tissues, where they prime adaptive immune response as well (3). The PRR-triggered responses of DCs are tightly controlled by a number of regulatory mechanisms, among them the mammalian target of rapamycin (mTOR) signaling pathway has received great attention lately.

Numerous studies have demonstrated that the mTOR pathway plays a central role in coordinating the development, differentiation and function of various immune cells including DCs (4). Besides, the mTOR pathway was found to be involved in the replication of a number of RNA and DNA viruses, which utilize host signaling pathways to enhance viral mRNA translation (5). mTOR is an evolutionarily conserved serine/threonine kinase that in response to environmental cues and intracellular signals controls cell growth, proliferation, metabolism and immune cell function. The mTOR protein is known to function as a component of two distinct multiprotein complexes: the rapamycin-sensitive mTOR complex 1 (mTORC1) and rapamycin-resistant mTORC2. Activation of mTORC1 leads to the phosphorylation of p70S6 kinase (p70S6K) and eukaryotic translation initiation factor 4E-binding protein 1 (4E-BP1), which promotes ribosome assembly and translation. Active mTORC2 phosphorylates multiple proteins including Akt, which couples extracellular signals to mTORC1 activation and other pathways controlling cell proliferation and survival (6).

The role of mTOR in DC biology has been broadly studied at both cellular and organism level using mTOR specific inhibitors (7). The immunosuppressive drug rapamycin is a potent allosteric mTOR inhibitor, which upon forming a complex with the intracellular FK506-binding protein 12 (FKBP12) inhibits mTORC1 downstream signaling while not affecting mTORC2 activity (8). It has been demonstrated first in plasmacytoid DCs (pDCs), which are the major cells in antiviral defense, that rapamycin pre-conditioning suppressed TLR9-induced type I interferon (IFN) production by both mouse and human pDCs (9). Rapamycin was also shown to inhibit the TLR9-induced release of type I IFNs from mouse myeloid DCs and Fms-like tyrosine kinase 3 ligand-dependent DCs as well (10). Interestingly, mTOR inhibition by rapamycin has been found to differentially regulate the cell surface TLR- and CD40 ligand-stimulated production of inflammatory cytokines and allogeneic T cell stimulatory capacity of human monocyte-derived DCs (moDCs) and CD1c + conventional DCs (cDCs) (11). Furthermore, inhibition of mTOR decreased the survival and increased the apoptosis of differentiating moDCs, while did not interfere with the differentiation of CD1c + cDCs. The second generation mTOR inhibitors such as AZD8055 and AZD2014, target the ATP binding site in the mTOR kinase domain, thus are able to inhibit the activity of both mTOR complexes. Rapamycin and the dual mTORC1/2 mTOR kinase inhibitor AZD2014 have been shown to reduce the generation of murine DCs from bone-marrow (BM-DC) (12). AZD8055 was demonstrated to induce a tolerogenic phenotype in BM-DC, that was indicated by the suppressed expression of major histocompatibility complex II (MHC II) and costimulatory molecules as well as by the decreased capacity to induce CD4 + T cell proliferation upon TLR4 stimulation (13). Previously we have reported that mTOR is also essential to the endosomal TLR3-mediated secretion of type I and III IFNs of human moDCs and blood circulating CD1c + cDCs (14). Thus, as outlined above, a growing body of evidence indicates that mTOR signaling shapes TLR-mediated DC responses, however its ability to modulate RLR-mediated activation and effector function of human DCs has not been explored yet.

RLRs are cytosolic viral sensors, which upon recognition of viral replication intermediates trigger signaling cascades leading to the production of type I IFNs and inflammatory cytokines (15). RIG-I and melanoma differentiation-associated protein 5 (MDA5), two major members of RLRs were reported to have distinct ligand preferences: RIG-I primarily recognizes short RNA sequences with 5’ triphosphate groups, while MDA5 predominantly recognizes long dsRNAs (16). We have previously published that similarly to blood circulating cDCs, moDCs constitutively express RLRs to sense foreign nucleic acids (17, 18). On the contrary, RIG-I and MDA5 levels are very low in resting pDCs, but can be greatly upregulated upon endosomal TLR stimulation. These observations indicate that, although, RLRs are absent from resting pDCs, they are inducible upon viral infections, thus contribute to the late phase of type I IFN responses of pDCs (18).

Many molecules have been previously identified as a regulator of RLR-mediated antiviral and inflammatory responses such as LGP2 (19), NOD2 (20), NLRX1 (17) or TRIM29 (21); however, to the best of our knowledge, the involvement of mTOR in RLR signaling of human DCs has not been investigated yet. Therefore, the goal of the present study was to reveal whether mTOR signaling contributes to the RLR-induced cytokine responses and T cell stimulatory potential of human moDCs and pDCs.

## Materials and Methods

### Isolation and Culturing of Primary Human Cells

The collection of human heparinized leukocyte-enriched buffy coat samples complied with the guidelines of the Helsinki Declaration and was approved by National Blood Transfusion Service and the Regional and Institutional Ethics Committee of the University of Debrecen, Faculty of Medicine (OVSzK 3572-2/2015/5200, Hungary). Buffy coat were obtained from healthy blood donors and peripheral blood mononuclear cells (PBMC) were separated by Ficoll-Paque Plus (Amersham Biosciences, Uppsala, Sweden) gradient centrifugation.

Monocytes were purified from PBMCs by positive selection using magnetic cell separation with anti-CD14-conjugated microbeads (Miltenyi Biotec, Bergisch Gladbach, Germany). Freshly isolated cells were seeded in 24-well cell culture plates at a density of 1 × 10^6^ cells/ml in RPMI 1640 medium (Sigma-Aldrich, St. Louis, MO, United States) supplemented with 10% heat-inactivated FBS (Life Technologies Corporation, Carlsbad, CA, United States), 2 mM L-glutamine, 100 U/ml penicillin, 100 μg/ml streptomycin (all from Sigma-Aldrich), 80 ng/ml GM-CSF (Gentaur Molecular Products, London, United Kingdom) and 50 ng/ml IL-4 (PeproTech, Brussels, Belgium) for 5 days.

Human pDCs were isolated from PBMCs by positive selection using the human CD304 (BDCA-4/Neuropilin-1) MicroBead Kit (Miltenyi Biotec) then cultured in 96-well cell culture plates at a density of 1 × 10^5^ cells/200 μl in RPMI 1640 medium (Sigma-Aldrich) supplemented with 10% heat-inactivated FBS (Life Technologies Corporation), 2 mM L-glutamine, 100 U/ml penicillin, 100 μg/ml streptomycin (all from Sigma-Aldrich), and 50 ng/ml recombinant human IL-3 (PeproTech).

Allogenic, naive CD8 + T cells were isolated from PBMC using the human naïve CD8 + T cell isolation kit (Miltenyi Biotec), and were used for DC-T cell co-culture experiments as described below.

All cells were incubated at 37°C in 5% CO_2_ humidified atmosphere.

### GEN2.2 Cell Line

The human pDC cell line (GEN2.2) (22) used in our experiments was provided by Dr. Joel Plumas and Dr. Laurence Chaperot (Research and Development Laboratory, French Blood Bank Rhône-Alpes, Grenoble, France) and was deposited with the CNCM (French National Collection of Microorganism Cultures) under the number CNCMI-2938. GEN2.2 cells were grown on a layer of mitomycin C (Sigma-Aldrich)-treated murine MS5 feeder cells (Cat. No. ACC 441, Leibniz Institute DSMZ-German Collection of Microorganisms and Cell Cultures, Braunschweig, Germany) in RPMI 1640 medium (Sigma-Aldrich) supplemented with 10% heat-inactivated FBS (Life Technologies Corporation), 2 mM L-glutamine, 100 U/ml penicillin, 100 μg/ml streptomycin (all from Sigma-Aldrich) and 5% non-essential amino acids (Life Technologies Corporation). For experiments, the GEN2.2 cells were removed from the feeder layer and seeded on 24-well plates at a concentration of 5 × 10^5^ cells/500 μl in complete RPMI 1640 medium (Sigma-Aldrich). Cell lines were grown and incubated at 37°C in 5% CO_2_ humidified atmosphere.

### Cell Stimulation

Before stimulation of moDCs half of the medium was removed and replaced by fresh complete medium supplemented with rapamycin (Merck Millipore, Darmstadt, Germany) or AZD8055 (Selleckchem, Houston, TX, United States) at a final concentration of 100 nM. Medium containing DMSO (final concentration 0.001%) was used as control treatment. After a 2 h incubation cells were stimulated with 3p-hpRNA (InvivoGen, San Diego, CA, United States), a specific agonist of RIG-I or high molecular weight polyinosinic:polycytidylic acid (polyI:C-HMW; InvivoGen) complexed with the transfection reagent LyoVec^TM^ (InvivoGen), at a final concentration of 0.5 μg/ml and 1 μg/ml, respectively, for the indicated time periods.

To induce RIG-I/MDA5 expression in GEN2.2 cells and primary pDCs, cells were pre-treated with the TLR9 agonist CpG-A (Hycult Biotech, Uden, The Netherlands) for 16 h as described previously (17, 23). Thereafter, the cells were washed, re-seeded in fresh, complete RPMI 1640 medium supplemented with mTOR inhibitors then stimulated with 3p-hpRNA/LyoVec^TM^ or polyI:C-HMW/LyoVec^TM^ complexes as described above for moDCs.

For live virus infection moDCs and CpG-A pre-treated pDCs were infected with vesicular stomatitis virus (VSV) Indiana serotype (VR-1238, ATCC, Manassas, VA, United States) at a MOI of 1 for 18 h.

### DC- T Cell Co-culture

Following activation, moDCs and primary pDCs were washed twice with cell culture medium and then co-cultured with freshly isolated, allogeneic naïve CD8 + T cells in RPMI 1640 medium (Sigma-Aldrich) supplemented with 10% heat-inactivated FBS (Life Technologies Corporation), 2 mM L-glutamine, 100 U/ml penicillin, 100 μg/ml streptomycin (all from Sigma-Aldrich) in the presence of 1 μg/ml anti-human CD3 mAb (BD Biosciences, Franklin, Lakes, NJ, United States). For proliferation assays, T cells were previously labeled with 0.5 μM carboxyfluorescein succinimidyl ester (CFSE, Invitrogen) and seeded on 96-well round bottom plates at a ratio of 2 × 10^4^ DC: 2 × 10^5^ T cell in 200 μl complete RPMI 1640 medium. After 5 days of co-cultivation, CFSE fluorescence intensities of T cells were detected in the FL1 (530 ± 15 nm) channel on a BD FACSCalibur flow cytometer (BD Biosciences). For intracellular cytokine staining naïve CD8 + T cells were seeded on 48-well cell culture plates at a ratio of 1:10 (1 × 10^5^ DCs; 1 × 10^6^ T cells) in 500 μl complete RPMI 1640 medium and after 6 days of co-cultivation were restimulated with 0.1 μg/ml phorbol myristate acetate and 1 μg/ml ionomycin (both from Sigma-Aldrich) in the presence of the protein transport inhibitor monensin (BD Biosciences) for 5 h. Thereafter, the cells were stained with anti-CD8-FITC (clone SK1) and isotype matched control antibody (both from BioLegend, San Diego, CA, United States), fixed and permeabilized by the BD Cytofix/Cytoperm solution (BD Biosciences). Cells were then labeled with APC-conjugated anti-IFN-y (clone 4S.B3) and anti-Granzyme B (clone QA18A28) antibodies and their isotype matched control antibodies (all from BioLegend). Fluorescence intensities were measured with FACSCalibur flow cytometer (BD Biosciences) and data were analyzed with FlowJo software (TreeStar, Ashland, OR, United States).

### Flow Cytometric Analysis of DCs

Phenotypical analysis of moDCs and GEN2.2 cells was performed by multicolor flow cytometry using anti-CD40-FITC (clone M5E2), anti-CD80-FITC (clone 2D10), anti-HLA-ABC-FITC (clone W6/32), anti-CD86-PE (clone IT2.2), anti-HLDA-DQ-PE (clone HLADQ1), anti-CD83-PeCy5 (HB15e), and isotype-matched control antibodies (all from BioLegend). Cell viability was assessed by 7-aminoactinomycin D (7-AAD; Sigma–Aldrich) staining for 15 min immediately before flow cytometric analysis. Fluorescence intensities were measured with FACSCalibur flow cytometer (BD Biosciences) and data were analyzed with FlowJo software (TreeStar).

### Western Blotting

For western blotting 5 × 10^5^ cells were lysed in Laemmli buffer, heated at 100°C for 10 min, separated on 7.5 or 10% SDS-PAGE then electro-transferred to nitrocellulose membranes (Bio-Rad Laboratories GmbH, Munich, Germany). Non-specific binding sites were blocked with 5% non-fat dry milk diluted in TBS Tween buffer (50 mM Tris, 0.5 M NaCl, 0.05% Tween-20, pH 7.4). Membranes were probed with the anti-RIG-I (clone D14G6), anti-MDA5 (clone D74E4), anti-phospho-Akt (Ser473; clone D9E), anti-Akt (clone 11E7), anti-phospho-p70S6 kinase (Thr389; clone D5U10) and anti-p70S6 kinase (clone 49D7), anti-phospho-Tank-binding kinase 1 (TBK1; clone D1B4), anti-TBK1 (clone D52C2), anti-phospho-p38 (clone 28B10) and anti-p38 antibodies (catalog number 9212) (all from Cell Signaling, Danvers, MA, United States). Beta-actin was used as a loading control (clone C4; Santa Cruz Biotechnology). The bound primary antibodies were conjugated with anti-mouse or anti-rabbit horseradish peroxidase-conjugated secondary antibodies (GE Healthcare, Little Chalfont, Buckinghamshire, United Kingdom) at a dilution of 1:5000 and 1:10000 respectively and were visualized by the ECL system using SuperSignal West Pico or Femto chemiluminescent substrate (Thermo Fisher Scientific, Rockford, IL, United States) and X-ray film exposure. Densitometric analysis of immunoreactive bands was performed using Image Studio Lite Software version 5.2 (LI-COR Biosciences, Lincoln, NE, United States).

### Quantitative Real Time PCR

Total RNA was isolated from 5 × 10^5^ cells using Tri Reagent (Molecular Research Center, Inc., Cincinnati, OH, United States). 1 μg of total RNA was treated with DNase I (Thermo Fisher Scientific, Waltham, MA, United States) to exclude amplification of genomic DNA then reverse transcribed into cDNA using the High Capacity cDNA RT Kit of Applied Biosystems (Foster City, CA, United States). Gene expression assays were purchased from Thermo Fisher Scientific for IL-6, TNF, hexokinase 2 (HK2), lactate dehydrogenase A (LDHA), hypoxia-inducible factor 1-alpha (HIF1A), and from Integrated DNA Technologies (Coralville, IA, United States) for PPIA (cyclophilin A) and IFNA1. Quantitative PCR was performed using the ABI StepOne Real-Time PCR System (Applied Biosystems) and cycle threshold values were determined using the StepOne v2.1 Software (Applied Biosystems). The relative amount of mRNA was obtained by normalizing to the PPIA housekeeping gene in each experiment.

### Assessment of Cytokines and Lactic Acid in Cell Supernatants

Cell culture supernatants were collected at the indicated time points. IFN-α levels were measured by the VeriKine^TM^ Human Interferon Alpha ELISA kit (PBL Interferon Sources, Piscataway, NJ, United States), whereas TNF and IL-6 levels were determined by the BD OptEIA human ELISA kits (BD Biosciences) according to the manufacturer’s instructions. Lactate production of the cells was detected using the Glycolysis Cell-Based Assay Kit (Cayman Chemical, Ann Arbor, Michigan, United States) according to the manufacturer’s protocol. Absorbance measurements were carried out by a Synergy HT microplate reader (Bio-Tek Instruments, Winooski, VT, United States) at 450 nm for cytokine detection and at 490 nm for lactate assay.

### Statistical Analysis

Statistical analysis was performed using ANOVA, followed by Bonferroni *post hoc* test by GraphPad Prism v.6. software (GraphPad Software Inc., La Jolla, CA, United States). Differences were considered to be statistically significant at *p* < 0.05.

## Results

### The mTOR Pathway Is Activated by RLR-Mediated Stimuli in moDCs

It is well known that TLR ligands activate mTORC1 and mTORC2 in innate immune cells (10, 11); however, whether mTOR signaling is integrated in the RLR signaling pathway of human DCs has not been investigated yet. To our studies, we used moDCs, which are closely related to inflammatory DCs and represent the best-studied model for human DC biology and for immunotherapy using DC vaccines against infectious diseases or cancer (24, 25). In order to analyze the role of mTOR, moDCs were pre-treated with rapamycin, an mTORC1-specific inhibitor, or AZD8055, which inhibits the activity of both mTORC1 and mTORC2, at clinically relevant doses prior to any other stimulation. Based on our previous publication the 100 nM concentration of rapamycin effectively inhibits mTOR signaling in moDCs while not affecting cell viability (14). After confirming that exposure to the same dose of AZD8055 did not alter cell viability (Supplementary Figures 1A,B, 2A,B), we have challenged the cells with 100 nM of both of the mTOR inhibitors for 2 h prior to RLR stimulation.

As a first step we tested whether mTOR inhibition affects the expression of the RLR receptors. Our results show that a 2 h treatment with rapamycin or AZD8055 does not alter the protein levels of RIG-I and MDA5 as compared to the solvent/vehicle control treated cells (Supplementary Figures 3A,B).

**FIGURE 1:**
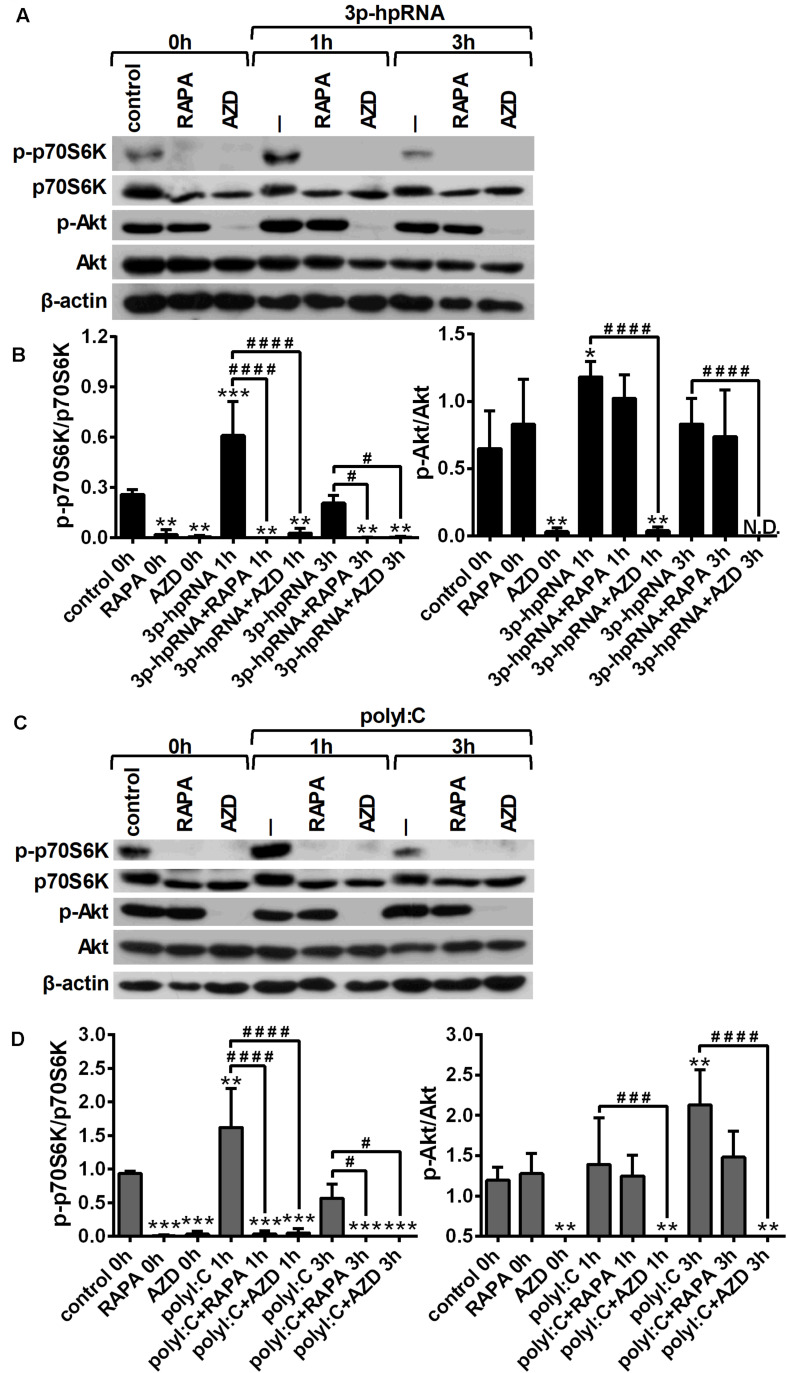
RLR stimulation increases mTORC1 and mTORC2 activity in moDCs that is effectively inhibited by rapamycin and AZD8055 pre-treatment. Immature moDCs were pre-treated with vehicle control, 100 nM rapamycin (RAPA) or 100 nM AZD8055 (AZD) for 2 h then stimulated with 3p-hpRNA (0.5 μg/ml) **(A,B)** or polyI:C (1 μg/ml) **(C,D)** for different time periods. Kinetics of p70S6K and Akt phosphorylation were determined by western blotting. **(A,C)** Representative blots are shown. **(B,D)** Bar graphs represent the mean ± SD of at least 3 independent experiments. *p < 0.05, **p < 0.01, ***p < 0.01 vs. control; ^#^*p* < 0.05, ^###^*p* < 0.001, ^####^*p* < 0.0001. ND, not determined.

To investigate whether RLR signaling drives mTOR activation in moDCs, we have analyzed the phosphorylation of p70S6K (Thr389), a major substrate of mTORC1 and Akt (Ser 473), the downstream target of mTORC2. Thus, 5-day moDCs were pre-treated with the mTOR inhibitors for 2 h and then stimulated with the RIG-I agonist 3p-hpRNA for different time periods (Figures 1A,B). Our results show that RIG-I stimulation significantly increased the phosphorylation of p70S6K showing a peak at 1 h of activation. Phosphorylation of p70S6K was markedly inhibited in the cells pre-treated with rapamycin or AZD8055. The phosphorylation of Akt at Ser473 was slightly but significantly increased upon RIG-I activation (Figures 1A,B) that was effectively inhibited by AZD8055. In parallel experiments, moDCs were stimulated with polyI:C (Figures 1C,D), which in complex with the transfection reagent LyoVec is a ligand for both cytoplasmic RIG-I and MDA5. Nevertheless, studies reported that the polyI:C/LyoVec complex is preferentially recognized by MDA5 (26). Following stimulation with polyI:C the phosphorylation of both p70S6K and Akt was significantly increased. Rapamycin inhibited the phosphorylation of only p70S6K, whereas AZD8055 efficiently abrogated the phosphorylation of p70S6K and Akt both in resting and activated cells. Thus, our results demonstrate that RLR stimuli increase the activity of mTOR signaling, which can be effectively repressed by the mTORC1 specific inhibitor rapamycin and the dual mTORC1/2 inhibitor AZD8055 (Figures 1C,D).

### mTOR Controls the RLR-Mediated Activation of moDCs

**FIGURE 2:**
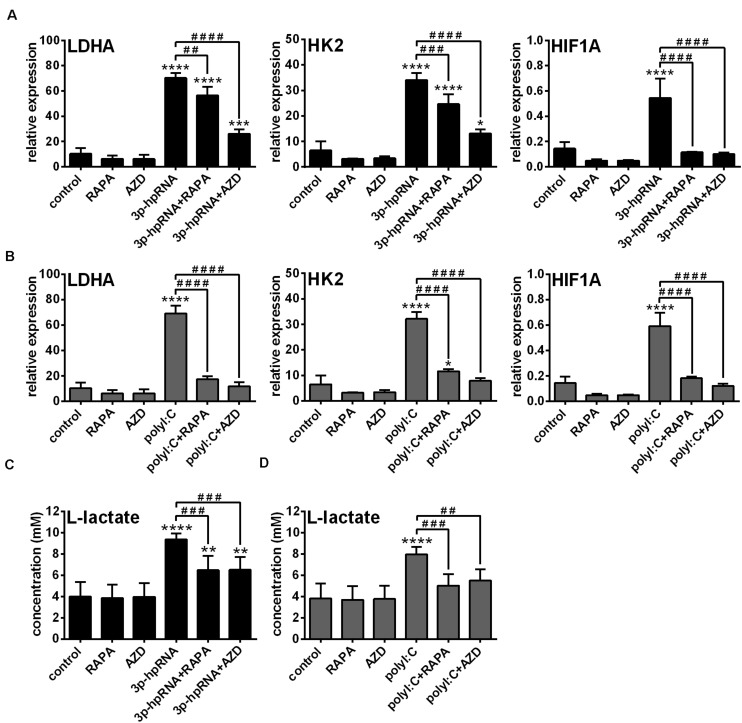
The RLR-mediated shift to glycolysis is impaired upon mTOR inhibition in moDCs. Immature moDCs were pre-treated with vehicle control, 100 nM rapamycin (RAPA) or 100 nM AZD8055 (AZD) for 2 h and then stimulated with 3p-hpRNA (0.5 μg/ml) **(A,C)** or polyI:C (1 μg/ml) **(B,D)** for 12 h. **(A,B)** The expression of *LDHA*, *HK2* and *HIF1A* was assessed at the mRNA level by real-time PCR. Bar graphs represent the mean ± SD of 4 independent experiments. **(C,D)** Lactate concentrations were measured from cell culture supernatants. Bar graphs represent the mean ± SD of 6 independent experiments. *p < 0.05, **p < 0.01, ***p < 0.01, ****p < 0.0001 vs. control; ^##^*p* < 0.01, ^###^*p* < 0.001, ^####^*p* < 0.0001.

Previously we have described that activation of moDCs by RIG-I results in a metabolic switch from oxidative phosphorylation to glycolysis, which promotes their type I IFN production and T cell priming ability (23). In contrast, we found that RIG-I-mediated antiviral responses of pDCs do not require glycolysis, implying that moDCs and pDCs have distinct metabolic profile upon RIG-I stimulation (23). Since it has been recognized that mTOR signaling supports the shift toward glycolysis upon TLR-mediated DC activation (27), we sought to reveal the role of mTOR in the glycolytic reprogramming of RLR-stimulated moDCs as well. Therefore, we examined the expression of glycolysis-related genes and levels of lactate in RLR-stimulated moDCs upon mTOR blockade. We found that both inhibitors abrogated the upregulation of *LDHA*, *HK2* and *HIF1A* (Figures 2A,B) and reduced the production of lactate (Figures 2C,D) in 3p-hpRNA- and polyI:C-stimulated cells. These results indicate that mTOR is essential to the RLR-mediated glycolytic changes of moDCs.

**FIGURE 3:**
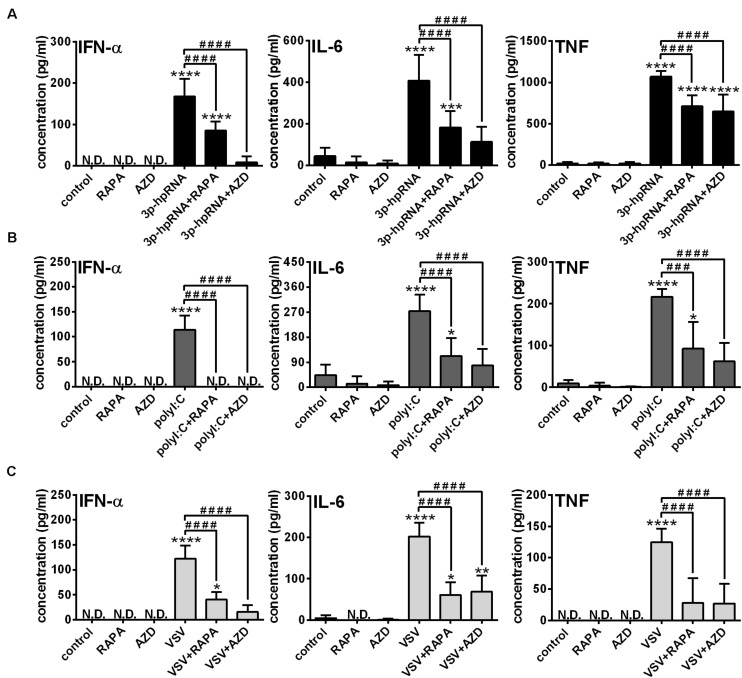
The RLR-mediated production of IFN-α and pro-inflammatory cytokines is decreased upon mTOR blockade in moDCs. Immature moDCs were pre-treated with vehicle control, 100 nM rapamycin (RAPA) or 100 nM AZD8055 (AZD) for 2 h and then stimulated with 3p-hpRNA (0.5 μg/ml) **(A)**, polyI:C (1 μg/ml) **(B)** or VSV (MOI 1) **(C)**. **(A–C)** IFN-α, IL-6 and TNF protein levels were measured by ELISA 24 h after 3p-hpRNA and polyI:C activation as well as 18 h after VSV stimulation. Data are shown as mean ± SD from 5 to 8 independent experiments. *p < 0.05, **p < 0.01, ***p < 0.01, ****p < 0.0001 vs. control; ^###^*p* < 0.001, ^####^*p* < 0.0001. ND, not determined.

Knowing that the shift to glycolysis, which provides energy for activation and cytokine production of cells, is mediated through mTOR, in the next step we investigated its involvement in the phenotypic and functional changes of RLR-stimulated moDCs. Following pre-treatment with rapamycin or AZD8055 cells were stimulated with 3p-hpRNA and polyI:C for 24 h then the expression of various membrane-bound molecules including the activation marker CD83, co-stimulatory molecules (CD80, CD86 and CD40) and MHC molecules (HLA-DQ and HLA-ABC) was measured by flow cytometry (Supplementary Figures 4A,B). Neither rapamycin nor AZD8055 altered the baseline and RLR-induced expression of the tested cell surface molecules. Next we analyzed the expression of type I IFNs, which are the major antiviral mediators as well as the induction of IL-6 and TNF pro-inflammatory cytokines both at the protein and mRNA levels. We found that resting moDCs do not secrete type I IFNs and only weekly produce inflammatory cytokines that is not affected by mTOR blockade. The RLR-triggered production of IFN-α, one of the major type I IFNs produced by moDCs and the secretion of IL-6 and TNF pro-inflammatory cytokines was significantly abrogated upon mTOR inhibition (Figures 3A,B). In line with that, we found that blocking of mTOR activity was able to decrease the RLR-driven upregulation of *IFNA1*, *IL-6* and *TNF* mRNA levels (Supplementary Figures 5A–D). To strengthen our observations, we also performed experiments with live virus. Following pre-treatment with rapamycin and AZD8055 moDCs were stimulated with VSV at a MOI of 1 for 18 h. As with the synthetic ligands, we found that mTOR blockade significantly reduced the VSV infection-induced production of IFN-α, IL-6 and TNF (Figure 3C).

**FIGURE 4:**
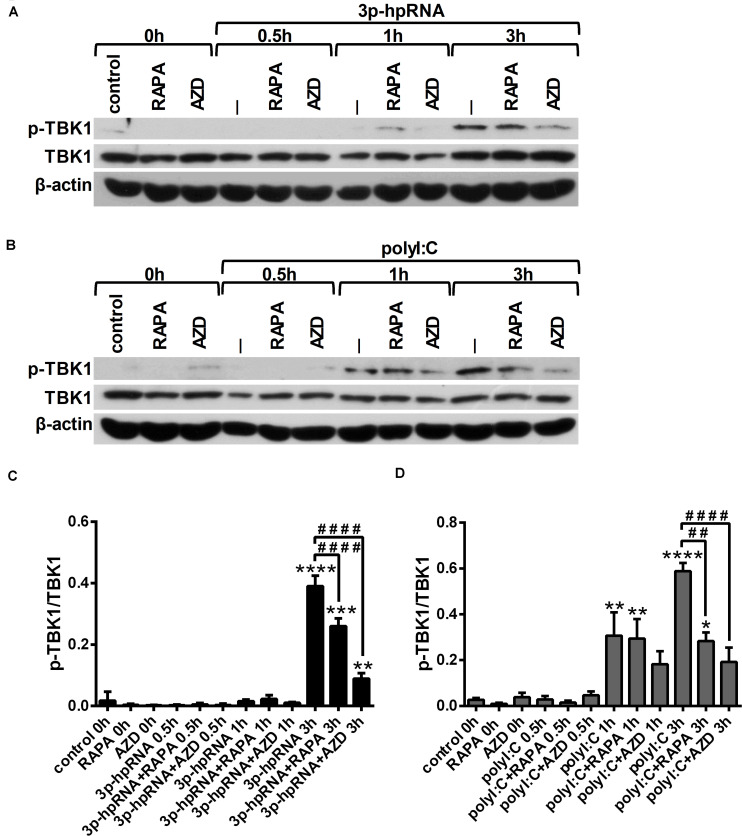
RLR stimulation enhances TBK1 activity in moDCs that is decreased by rapamycin and AZD8055 pre-treatment. Immature moDCs were pre-treated with vehicle control, 100 nM rapamycin (RAPA) or 100 nM AZD8055 (AZD) for 2 h then stimulated with 3p-hpRNA (0.5 μg/ml) **(A,C)** or polyI:C (1 μg/ml) **(B,D)** for different time periods. Kinetics of TBK1 phosphorylation were determined by western blotting. **(A,B)** Representative blots are shown. **(C,D)** Bar graphs represent the mean ± SD of at least 3 independent experiments. *p < 0.05, **p < 0.01, ***p < 0.01, ****p < 0.0001 vs. control; ^##^*p* < 0.01, ^####^*p* < 0.0001.

Since RLR signaling culminates in the activation of the serine/threonine-protein kinase TBK1, which is involved in the production of both pro-inflammatory and antiviral cytokines (28, 29), we analyzed TBK1 activity by detecting its phosphorylation at serine 172 (Figures 4A–D). Our results indicate that RLR stimulation increases the level of TBK1 phosphorylation, which can be significantly decreased upon mTOR inhibition. The mitogen activated protein kinase (MAPK) p38 was also reported to be essential to the RIG-I-mediated interferon production and activation of DCs (30). However, measuring the phosphorylation levels of p38 (Thr180/Tyr182), we did not observed any differences upon RLR stimulation or mTOR blockade (Supplementary Figures 6A–D).

All these data demonstrate that inhibition of mTORC1 as well as mTORC1/C2 did not restrained the phenotypic maturation of RLR-stimulated moDCs, while significantly reduced their cytokine responses probably through decreasing TBK1 activity.

### RLR Stimulation Enhances mTOR Activity in pDCs

To determine the importance of mTOR in RLR-mediated antiviral responses of other DC subsets next we focused our interest on pDCs, which are best known for their ability to produce large amounts of type IFNs as well as various pro-inflammatory cytokines in response to a viral infection (31). Due to the limiting number of circulating pDCs in the peripheral blood we performed some of our experiments with the human GEN2.2 pDC cell line, which shows similar phenotypic and functional properties to primary human pDCs (32, 33). Nevertheless, the cytokine profile and T cell stimulatory potential of pDCs were confirmed with primary human pDCs isolated from peripheral blood. Based on our previous findings moDCs express baseline levels of RLRs, while pDCs require a pre-treatment with the TLR9 agonist CpG-A to induce the expression of RIG-I and MDA5 (18). Therefore, GEN2.2 cells or primary pDCs were pre-treated with a low dose of CpG-A for 16 h then washed thoroughly prior to any other treatments. First, to investigate the role of mTOR in RLR-dependent signaling we examined whether mTOR blockade modifies the expression of RLR receptors in pDCs. As with moDCs, a 2 h treatment with rapamycin and AZD8055 did not induce any changes in the protein levels of RIG-I and MDA5 (Supplementary Figures 3C,D).

**FIGURE 5:**
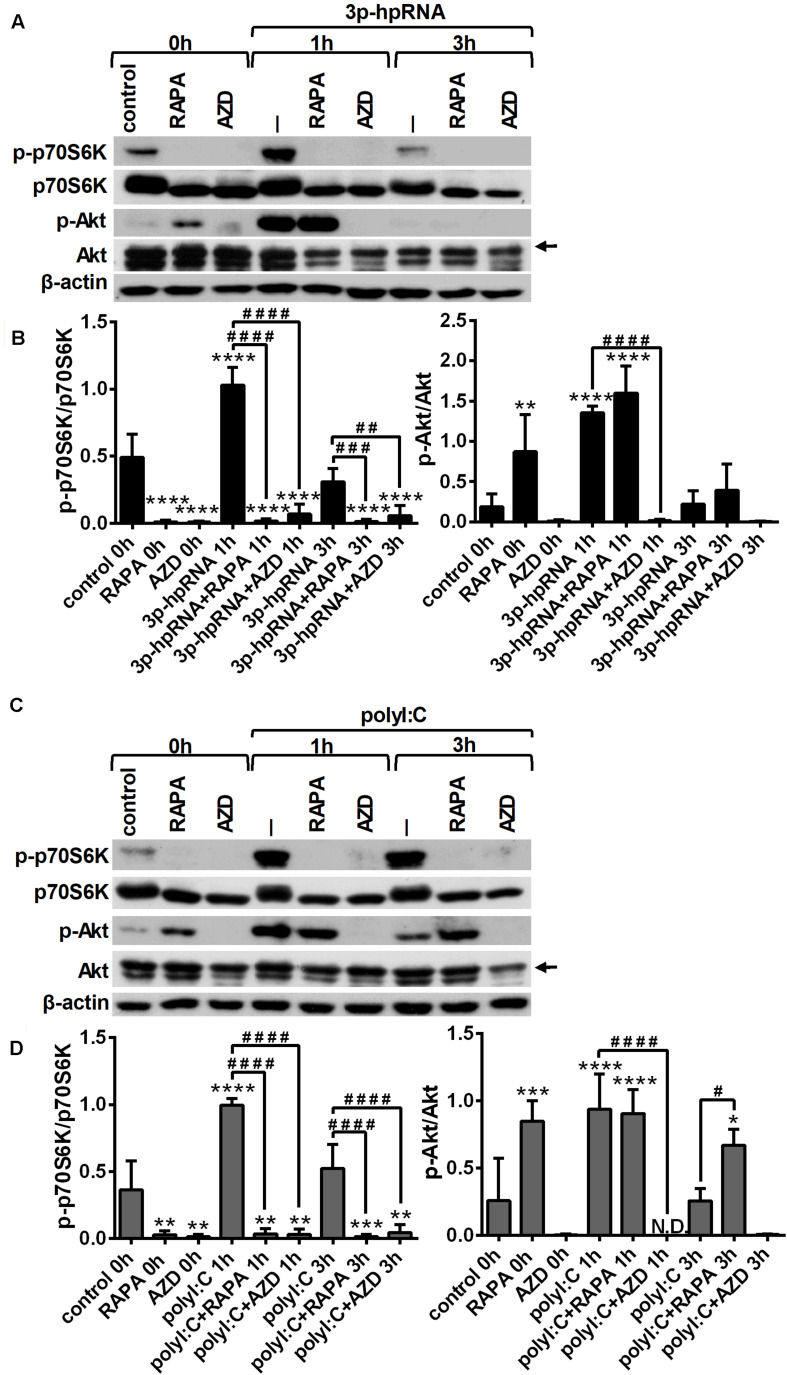
RLR stimulation increases mTORC1 and mTORC2 activity in GEN2.2 cells that can be effectively inhibited by rapamycin and AZD8055 pre-conditioning. GEN2.2 cells were pre-treated with vehicle control, 100 nM rapamycin (RAPA) or 100 nM AZD8055 (AZD) for 2 h and then stimulated with 3p-hpRNA (0.5 μg/ml) **(A,B)** or polyI:C (1 μg/ml) **(C,D)** in a time-dependent manner. Kinetics of p70S6K and Akt phosphorylation was determined by western blotting. **(A,C)** Representative blots are shown. **(B,D)** Bar graphs represent the mean ± SD from 4 independent experiments. Specific bands for Akt are indicated by arrows. *p < 0.05, **p < 0.01, ***p < 0.01, ****p < 0.0001 vs. control; ^#^*p* < 0.05, ^##^*p* < 0.01, ^###^*p* < 0.001, ^####^*p* < 0.0001. ND, not determined.

Next, we determined whether mTOR signaling affects the activation of RLRs in pDCs similarly to moDCs, therefore we analyzed the phosphorylation rate of p70S6K (Thr389) and Akt (Ser473), the downstream targets of mTORC1 and mTORC2, respectively. GEN2.2 cells were pre-conditioned with 100 nM rapamycin or AZD8055 for 2 h then stimulated with RLR agonist and the kinetics of Akt and p70S6K phosphorylation was determined by western blotting. The time-dependent analysis shows that similar to moDCs, stimulation of pDCs with 3p-hpRNA (Figures 5A,B) and polyI:C (Figures 5C,D) significantly increased the phosphorylation of p70S6K and Akt showing a peak at 1 h after challenge. Our results further demonstrate that pre-treatment with rapamycin successfully abrogated the phosphorylation of only p70S6K both in resting and activated cells, whereas the dual mTORC1/2 inhibitor, AZD8055 blocked the phosphorylation of both mTOR targets. We also found that pre-conditioning by rapamycin alone leads to an increase in Akt phosphorylation that can be explained by the loss of negative feedback loop from mTORC1 and p70S6K (34). These results are in line with data obtained in moDCs showing that mTOR activity is increased by RLR stimuli suggesting its involvement in RLR-mediated innate immune responses of human pDCs as well.

### mTOR Modulates the RLR-Triggered Maturation of pDCs

Because RLR engagement was found to activate mTOR signaling in pDCs, we were also curious how mTOR inhibition could impact the RLR-induced phenotypic and functional changes of pDCs. Pre-treatment with rapamycin or AZD8055 was followed by stimulation with 3p-hpRNA and polyI:C for 24 h and then the expression of the same panel of membrane-bound molecules as for moDCs was analyzed by flow cytometry (Supplementary Figures 7A,B). Similar to moDCs, mTOR blockade did not affect the baseline level or RLR-triggered expression of the investigated cell surface molecules. Interestingly, RLR stimulation increased significantly the expression of the CD83 activation marker, which was further enhanced by AZD8055 pre-treatment.

**FIGURE 6:**
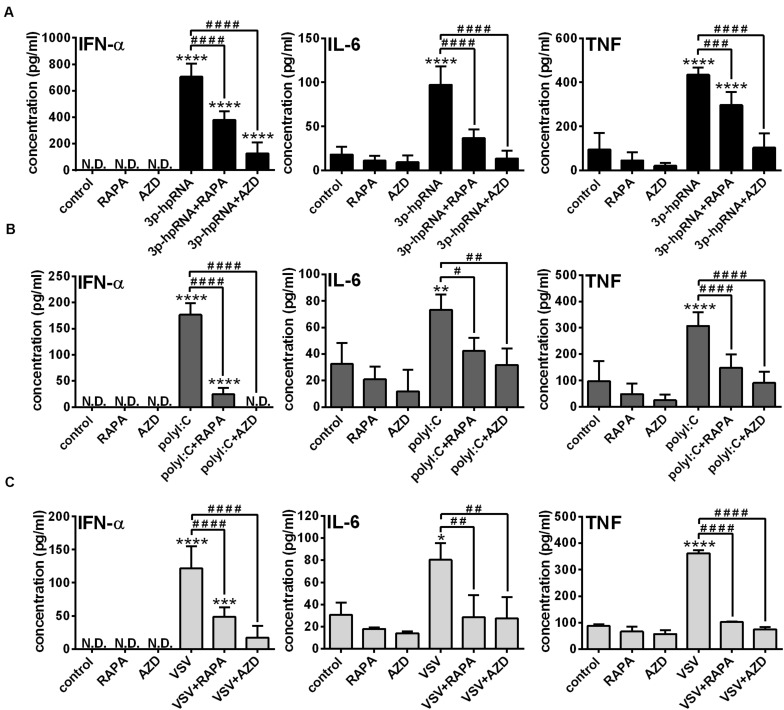
The RLR-mediated production of IFN-α and pro-inflammatory cytokines is decreased upon mTOR inhibition in GEN2.2 cells. GEN2.2 cells were pre-treated with vehicle control, 100 nM rapamycin (RAPA) or 100 nM AZD8055 (AZD) for 2 h then stimulated with 3p-hpRNA (0.5 μg/ml) **(A)**, polyI:C (1 μg/ml) **(B)**, or VSV (MOI 1) **(C)**. **(A–C)** IFN-α, IL-6 and TNF protein levels were measured by ELISA 6 h after 3p-hpRNA as well as polyI:C activation and 18 h after VSV stimulation. Data are shown as mean ± SD from 4 to 8 independent experiments. *p < 0.05, **p < 0.01, ****p < 0.0001 vs. control; ^#^*p* < 0.05, ^##^*p* < 0.01, ^###^*p* < 0.001, ^####^*p* < 0.0001. ND, not determined.

Next, to identify the impact of mTOR activity on the effector functions of pDCs, the secretion of IFN-α and pro-inflammatory cytokines was analyzed in RLR-activated cells upon pre-incubation with mTOR inhibitors. In GEN2.2 cells, inhibition of mTORC1 with rapamycin or mTORC1/2 with AZD8055 alone neither induced IFN-α production nor altered the secretion of IL-6 and TNF. Consistent with the data obtained with moDCs, 3p-hpRNA (Figure 6A) and polyI:C (Figure 6B) significantly increased the production of IFN-α, IL-6 and TNF cytokines, which was prevented upon pre-treatment with rapamycin or AZD8055. To further confirm our results, we also tested the cytokine production of live virus infected cells and found that inhibition of mTOR impaired profoundly the VSV-induced release of IFN-α, IL-6 and TNF as well (Figure 6C).

**FIGURE 7:**
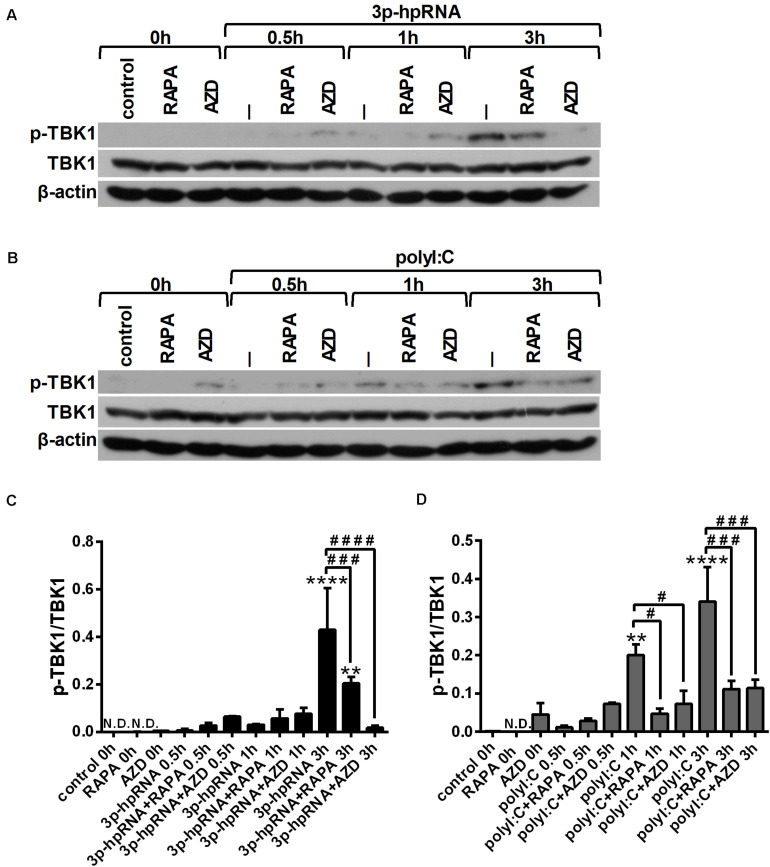
RLR stimulation increases the phosphorylation of TBK1 that is reduced by rapamycin and AZD8055 pre-treatment in GEN2.2 cells. Cells were pre-treated with vehicle control, 100 nM rapamycin (RAPA) or 100 nM AZD8055 (AZD) for 2 h then stimulated with 3p-hpRNA (0.5 μg/ml) **(A,C)** or polyI:C (1 μg/ml) **(B,D)** for different time periods. Kinetics of TBK1 phosphorylation were determined by western blotting. **(A,B)** Representative blots are shown. **(C,D)** Bar graphs represent the mean ± SD of at least 3 independent experiments. **p < 0.01, ****p < 0.0001 vs. control; ^#^*p* < 0.05, ^###^*p* < 0.001, ^####^*p* < 0.0001. ND, not determined.

Similar to moDCs, we also analyzed the phosphorylation levels of TBK1 (Figures 7A–D) and p38 (Supplementary Figures 8A–D) to reveal the mechanism underlying the effects of mTOR inhibitors on RLR-mediated cytokine production of pDCs. In line with our observation on moDCs, we found that RLR-mediated activation of TBK1 was significantly inhibited in the presence of rapamycin or AZD8055 in GEN2.2 cells as well, whereas the phosphorylation of p38 was not affected.

**FIGURE 8:**
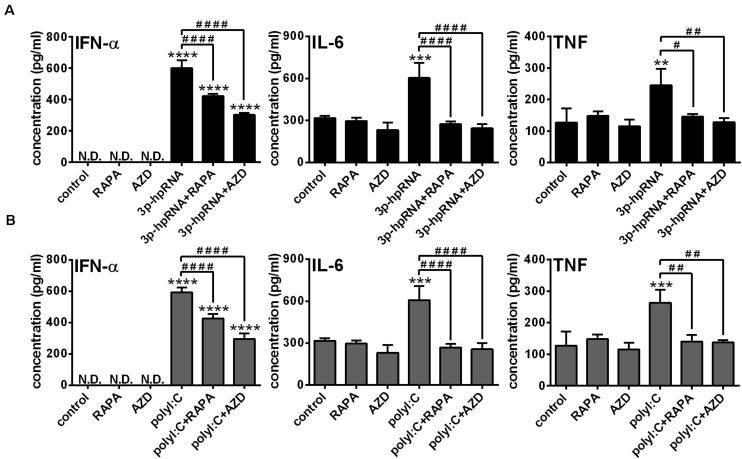
The RLR-stimulated secretion of IFN-α and pro-inflammatory cytokines is abolished upon mTOR blockade in primary human pDCs. Primary pDCs were pre-treated with vehicle control, 100 nM rapamycin (RAPA) or 100 nM AZD8055 (AZD) for 2 h and then stimulated with 3p-hpRNA (0.5 μg/ml) **(A)** or polyI:C (1 μg/ml) **(B)**. **(A,B)** IFN-α, IL-6 and TNF protein levels were measured by ELISA 6 h after stimulation. Data are shown as mean ± SD from 3 independent experiments. **p < 0.01, ***p < 0.01, ****p < 0.0001 vs. control; ^#^*p* < 0.05, ^##^*p* < 0.01, ^####^*p* < 0.0001. ND, not determined.

To further confirm our findings obtained with the human pDC cell line, we conducted experiments with primary human pDCs from healthy blood donors as well. Primary pDCs were cultured in the presence or absence of mTOR inhibitors for 2 h and then stimulated with 3p-hpRNA and polyI:C for 6 h. In line with our observations on GEN2.2 cells, inhibition of mTORC1 with rapamycin or mTORC1/2 with AZD8055 alone did not influence the levels of IFN-α, IL-6 and TNF. Furthermore, mTOR blockade decreased the 3p-hpRNA stimulated (Figure 8A) and polyI:C triggered (Figure 8B) release of IFN-α as well as the secretion of IL-6 and TNF pro-inflammatory cytokines. These data demonstrate that both selective mTORC1 and dual mTORC1/2 inhibition affects similarly the RLR-triggered cytokine responses of pDCs.

### Rapamycin Is a Stronger Inhibitor of DC-Mediated T Cell Responses Then the Dual mTORC1/mTORC2 Kinase Inhibitor AZD8055

**FIGURE 9:**
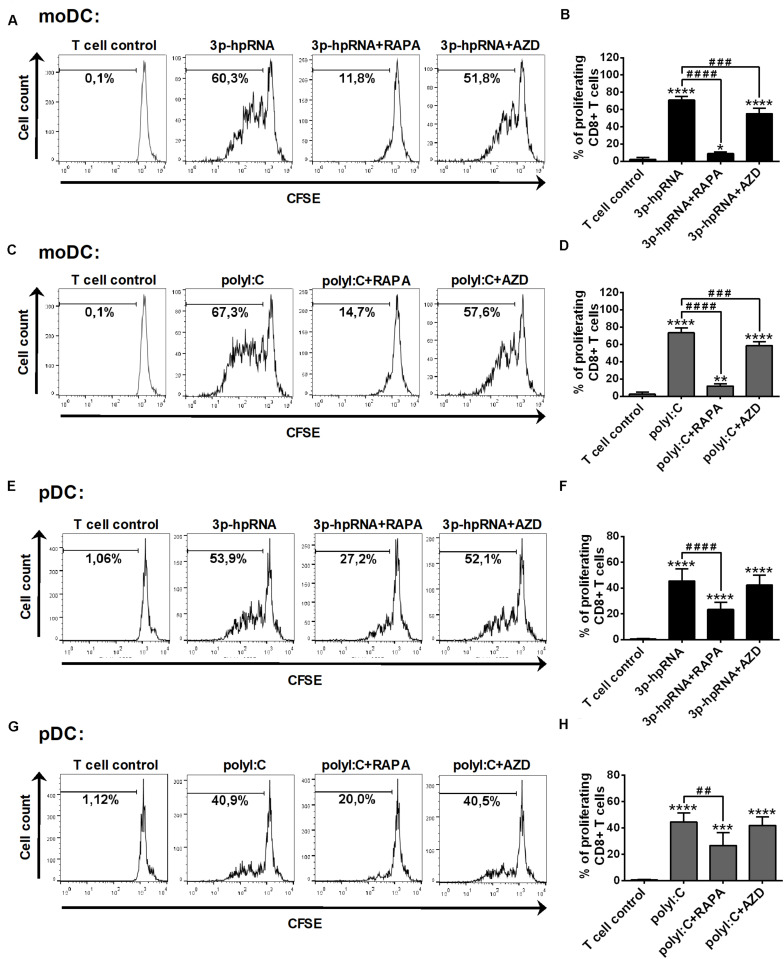
Rapamycin but not AZD8055 effectively inhibits the ability of RLR-stimulated moDCs and primary pDCs to induce proliferation of naive T cells. CFSE-labeled naïve CD8 + T cells were co-cultured with allogeneic moDCs **(A–D)** and primary pDCs **(E–H)** pre-treated with the indicated reagents. After 5 days of co-cultivation, cell division was measured by flow cytometry. **(A,C,E,G)** Representative histograms are shown where numbers indicate the percentage of viable dividing T cells. In **A** and **C** T cell controls are the same, since representative dot plots originate from the same set of experiment from one single donor. **(B,D,F,H)** Bar graphs represent the mean ± SD of at least 6 independent experiments. *p < 0.05, **p < 0.01, ****p < 0.0001 vs. control; ^###^*p* < 0.001, ^####^*p* < 0.0001. AZD: AZD8055, RAPA: rapamycin.

Having established that mTOR blockade impaired the production of type I IFNs and pro-inflammatory cytokines induced by RLR stimulation, next, we sought to investigate the capacity of moDCs and primary pDCs to promote T cell responses upon mTOR inhibition. Therefore, allogeneic naïve CD8 + T cells were co-cultured with DCs treated as described above then the proliferation and cytokine production of T cells were measured by flow cytometry. In these experiments, DCs were stimulated with synthetic ligands instead of live virus, since we cannot exclude the possibility that T cells get infected with VSV in the co-culture. Our results demonstrate that moDCs treated with 3p-hpRNA (Figures 9A,B) or polyI:C (Figures 9C,D) induced significant T cell proliferation, which was inhibited when moDCs were pre-treated with rapamycin prior to RLR stimulation. On the contrary, the active site inhibitor AZD8055 was much weaker in inhibiting the ability of moDCs to stimulate T cell proliferation. Similarly, rapamycin effectively reduced, whereas AZD8055 did not affect RLR-stimulated pDCs (Figures 9E–H) in their capacity to induce T cell proliferation.

**FIGURE 10:**
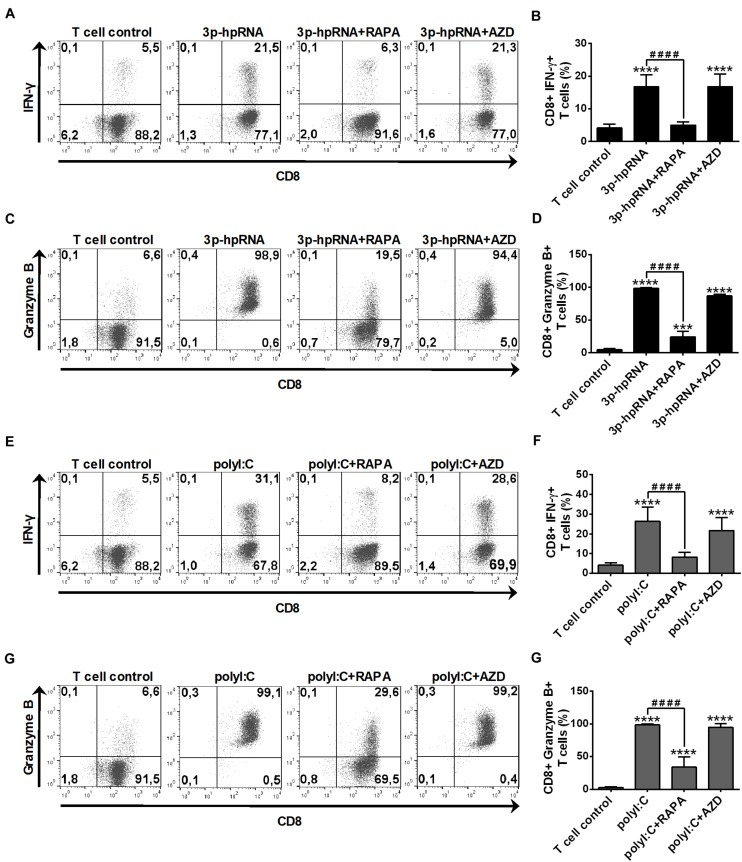
Rapamycin but not AZD8055 decreased the T cell activating capacity of RLR-activated moDCs. Naïve CD8 + T cells were co-cultured with allogeneic moDCs pre-treated with the indicated reagents. After 6 days of co-cultivation T cells were stimulated with phorbol myristate acetate (0.1 μg/ml) and ionomycin (1 μg/ml) in the presence of monensin for 5 h. IFN-y and Granzyme B production of CD8 + T cells was measured by intracellular cytokine staining using flow cytometry. **(A,C,E,G)** Data from one representative experiment are shown. Numbers in quadrants indicate percent cells in each. In **A** and **E** as well as in **C** and **G** T cell controls are the same, since representative dot plots originate from the same set of experiment from one single donor. **(B,D,F,H)** Bar graphs represent the mean ± SD of 6 independent experiments. ***p < 0.01, ****p < 0.0001 vs. control; ^####^*p* < 0.0001. AZD: AZD8055, RAPA: rapamycin.

**FIGURE 11:**
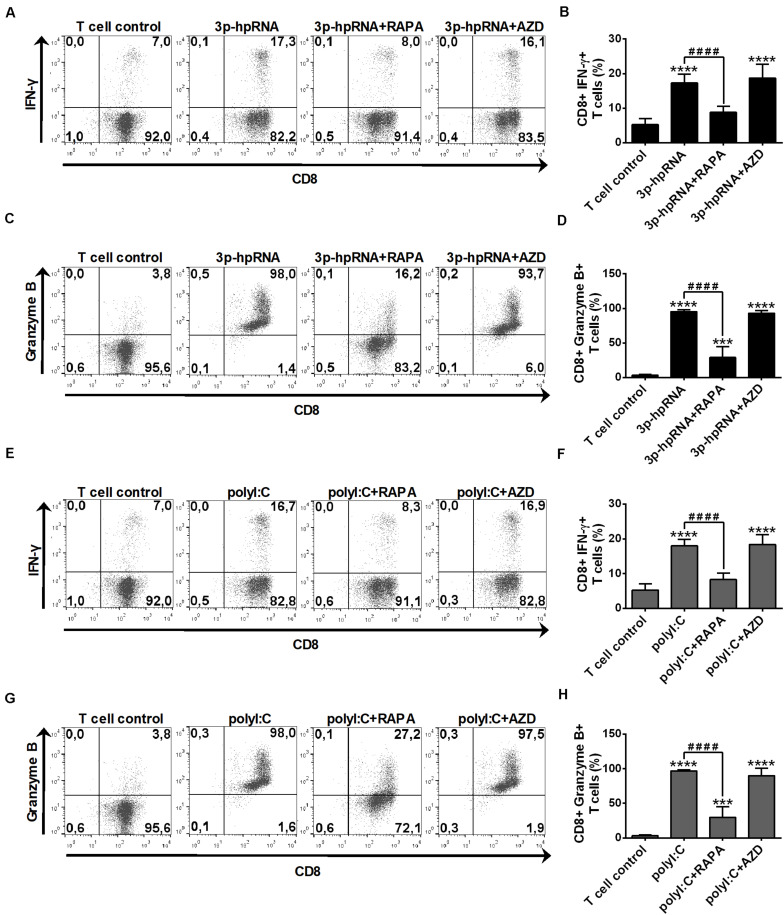
The T cell activating capacity of RLR-stimulated primary human pDCs is decreased by rapamycin but not AZD8055 pre-treatment. Naïve CD8 + T cells were co-cultured with allogeneic primary human pDCs pre-treated with the indicated reagents. After 6 days of co-cultivation T cells were stimulated with phorbol myristate acetate (0.1 μg/ml) and ionomycin (1 μg/ml) in the presence of monensin for 5 h. IFN-y and Granzyme B production of CD8 + T cells was measured by intracellular cytokine staining and flow cytometry. **(A,C,E,G)** Data from one representative experiment are shown. Numbers in each quadrant represent the percentage of cells. In **A** and **E** as well as in **C** and **G** T cell controls are the same, since representative dot plots originate from the same set of experiment from one single donor. **(B,D,F,H)** Bar graphs represent the mean ± SD of 6 independent experiments. ***p < 0.01, ****p < 0.0001 vs. control; ^####^*p* < 0.0001. AZD: AZD8055, RAPA: rapamycin.

In parallel experiments, we also analyzed the effector functions of DC-stimulated CD8 + T cells by measuring intracellular levels of the pro-inflammatory cytokine IFN-y and the cytotoxic protein Granzyme B. MoDCs activated with 3p-hpRNA (Figures 10A–D) or polyI:C (Figures 10E–H) triggered the production of high levels of IFN-y and Granzyme B in T cells. Intriguingly, the T cell activating capacity of moDCs was significantly impaired upon rapamycin pre-treatment, whereas was not affected by AZD8055 pre-conditioning. We obtained very similar results with pDCs, the ability of which to promote IFN-y and Granzyme B production in T cells was diminished upon rapamycin but not by AZD8055 pre-treatment (Figures 11A–H). All these observations reveal that mTORC1 but not mTORC1/C2 inhibition is required to inhibit effectively the T cell stimulatory potential of human DCs.

## Discussion

DCs are potent antigen presenting cells and thus play a critical role in orchestrating innate and adaptive immune responses against viruses (35). For the reason that many viruses replicate in the cytoplasm, host cells have had to evolve mechanisms to detect pathogens intracellularly as well. Upon reaching the cytoplasm, viral nucleic acids can be sensed by RLRs, which are key elements of host defense by triggering innate immune responses to control and eliminate viral infections (36). Following activation RIG-I or MDA5 initiate a cascade of downstream signaling events leading to the production of type I IFNs and pro-inflammatory cytokines that altogether elicit an antiviral state to eradicate the virus from the host (37). In the last decade, mTOR has been emerged as a central regulator of TLR-mediated DC maturation and function, how it affects RLR-dependent antiviral signaling of human DCs has not been explored yet.

Previous results showed that the PI3K/mTOR pathways regulates various aspects of DC biology including differentiation, maturation, cytokine production and T cell stimulatory potential of DCs as well (38). The very first study describing a link between TLR signaling and mTOR activity in DCs showed that stimulation of pDCs with the TLR9 ligand CpG-A enhanced the phosphorylation of p70S6K, the major downstream target of mTORC1, which was inhibited upon rapamycin pre-treatment (9). Subsequent studies also demonstrated that various TLR ligands are able to induce p70S6K phosphorylation, which was abrogated by rapamycin in both human and murine DCs. TLR ligands also activate mTORC2 in different innate immune cells such as monocytes, macrophages and DCs (39–42). For instance, studies have demonstrated that stimulation of mouse BM-DCs with the TLR4 agonist lipopolysaccharide induced the phosphorylation of Akt on serine 473, which is a commonly used readout for mTORC2 activity (42, 43). Moreover, several viruses are also able to activate the PI3K/Akt/mTOR signaling to promote cell survival and inhibit apoptosis (44, 45). It has been published that infection of lung epithelial cells with Sendai virus, which is primarily recognized by RIG-I but can also be detected by MDA5 and TLR7, induces the phosphorylation of several mTOR kinase substrates suggesting increased mTOR activity (46). Previous studies also confirmed that mTORC1 is a key factor in regulating the replication of coronaviruses, the replication intermediates of which are suggested to be recognized by RLRs (47, 48). Here we demonstrate that similar to TLR triggering, RLR stimulation of human DCs also results in the activation of the mTOR signaling pathway. We found that activation of moDCs and pDCs with RIG-I/MDA5 specific ligands significantly increased p70S6k and Akt phosphorylation, which reflects enhanced mTORC1 and mTORC2 activity, respectively.

The mTOR signaling network has been found to play a central role in coordinating DC metabolic reprogramming in response to microbial stimuli (49, 50). We have previously reported that RIG-I stimulation of moDCs but not of pDCs is also accompanied by an increase in glycolysis (23), thus we have tested how mTOR inhibition affects the RLR-driven metabolic shift toward glycolysis in moDCs. Both rapamycin and AZD8055 reduced significantly the RLR-mediated release of lactate and upregulation of glycolysis related genes that reflects decreased glycolytic rate upon mTOR blockade.

There are several and sometimes controversial reports about the ability of mTOR to control the phenotypic profile and cytokine producing properties of different DC subtypes. In pDCs and Fms-like tyrosine kinase 3 ligand-dependent DCs inhibition of mTOR or its downstream target p70S6K have been shown to impair TLR9-mediated type I IFN production (9, 10). In moDCs, short term blockade of mTOR leads to a decrease in their inflammatory cytokine production such as IL-6 and TNF after stimulation of various cell surface TLRs. On the contrary, rapamycin pre-treatment of CD1c + cDCs increased TLR4-induced production of IL-6, while did not affect TNF secretion (11). In addition, we have previously reported that mTOR is essential to the production of type I and III IFNs in both moDCs and CD1c + cDCs following endosomal TLR3 stimulation (14). A possible link between mTOR and RLR signaling was suggested in a previous study, in which rapamycin pre-treatment was reported to decrease the polyI:C-induced secretion of IFN-α in mouse macrophages lacking the TLR3 adaptor molecule TRIF (10). The results obtained from our current study clearly demonstrate that mTOR also acts as a crucial regulator of RLR-mediated cytokine responses in human DCs since both rapamycin and AZD8055 decreased significantly the RLR-mediated secretion of IFN-α, IL-6 and TNF of moDCs and pDCs. Consistent with that, upon infection of DCs with live replicating VSV, which is known to be specifically recognized by RIG-I rather than by MDA5 (15), we also observed impaired cytokine production in the presence of mTOR inhibitors. In line with our data, other studies previously reported that mTOR inhibition reduced the VSV-triggered production of IFN-α in Flt3-ligand-dependent DCs (10) and decreased the type I and III IFN production of human lung epithelial cells in response to Sendai virus infection (46). To reveal how mTOR regulates the RLR-mediated cytokine responses of DCs we have measured the activity of TBK1, which leads to the activation of nuclear factor-kappa B (NF-kB) and interferon regulatory factor 3 (IRF3) transcription factors and eventually to the production of pro-inflammatory cytokines and type I IFNs (28, 29). Our results show that mTOR blockade reduces the RLR-triggered phosphorylation of TBK1, thus we suppose, that mTOR regulates the cytokine production of DCs via inhibiting the RLR-initiated phosphorylation of TBK1. These data demonstrate that mTOR signaling is critical for the antiviral cytokine response of various cell types including DCs.

In contrast to cytokine responses, we could not detect significant alterations in the phenotype of RLR-stimulated DCs upon mTOR inhibition. Nevertheless, previous studies including ours have reported changes in the cell surface molecule expression of TLR-triggered DCs. Previously we have found that the levels of CD83, a frequently used DC maturation marker, was increased in CD1c + cDCs but was unaltered in moDCs upon rapamycin pre-conditioning (14). In line with these data, our present study demonstrates that RLR-mediated upregulation of CD83 is unaffected in moDCs by rapamycin or AZD8055 pre-conditioning. Interestingly, in RLR-stimulated pDCs rapamycin did not affect while AZD8055 increased the expression of CD83. All the other tested cell surface molecules, including costimulatory, MHC class I and II proteins were not affected by mTOR inhibition either in moDCs or pDCs indicating that mTOR blockade does not interfere with the RLR-triggered phenotypic changes of human DCs. All these data suggest that mTOR-mediated effects on the phenotype of DCs vary greatly depending on the cell type, mode of activation and type of receptor stimulated.

Data from many studies indicate that mTOR is also involved in the antigen presentation by DCs and thereby able to modulate their ability to stimulate effector T cell responses (38). The cytokine milieu created by DCs in response to viral infection acts in collaboration with the antigenic signal to drive the differentiation and survival of antiviral CD8 + T cells (51). Importantly, type I IFNs play a major role in T cell responses against viral infected cells by allowing the clonal expansion and prolonging the survival of effector CD8 + T cells (52). Since mTOR inhibition decreased the RLR-driven production of IFN-α by DCs, we were curious how their CD8 + T cell stimulatory capacity is affected. Our co-culture experiments demonstrate that rapamycin effectively reduces the ability of RLR-stimulated moDCs and pDCs to prime CD8 + T cell proliferation. Interestingly, the dual mTOR kinase inhibitor AZD8055 only slightly decreased the T cell proliferation capacity of RLR-stimulated moDCs, while not affecting pDCs. In line with our observation another study demonstrated that rapamycin is a more potent inhibitor of BM-DC-mediated CD8 + T cell proliferation then the dual mTOR kinase inhibitor AZD2014, which must be applied in a dose 20-fold higher than rapamycin to achieve a similar inhibitory effect (12). Nevertheless, it must be noted that higher doses of mTOR inhibitors show greater toxicity (53). In line with these observations we found that AZD8055 at doses greater than 100 nM decreased moDCs survival significantly (data not shown). Similarly, Torin-1, another ATP-competitive mTOR inhibitor was less effective than rapamycin to reduce the T cell stimulatory capacity of TLR4-stimulated BM-DCs (54). Moreover, AZD8055, but not rapamycin combined with an agonist CD40 antibody elicited synergistic antitumor response in a metastatic renal cell carcinoma model (55). Analyzing the DC-mediated effector functions of T cells, we also found that rapamycin significantly decreased, whereas AZD8055 did not affect the ability of RLR-stimulated DCs to induce IFN-γ and Granzyme B production by CD8 + T cells. This phenomenon could be possible explained by the loss of an mTORC2-mediated negative feedback loop that is suppressed by dual mTOR kinase inhibitors. Recently, the NPV-BEZ235 dual PI3K/mTOR kinase inhibitor was demonstrated to overactivate the mitogen-activated protein kinase kinase (MEK)/extracellular signal-regulated kinase (ERK) pathway in human pancreatic cancer cells (56). Stimulation of DCs via PRRs leads to the activation of various MAPKs and among them the MEK/ERK signaling pathway was demonstrated to act as a positive regulator of antigen presentation in moDCs. Interestingly, blockade of mTORC2 alone seems to enhance rather than inhibit the ability of DCs to promote T cell responses. For instance, absence of mTORC2 in cutaneous DCs elicited enhanced CD8 + T cell effector responses and augmented skin graft rejection (57). In addition, mTORC2 deficient mouse myeloid DCs exhibited enhanced T cell allostimulatory ability and increased capacity to expand IFN-γ- and IL-17-producing T cells following TLR and non-TLR stimulation (58). Though, the mechanism behind the increased immunogenicity of mTORC2-deficient DCs has not been revealed yet. Thus, further studies are needed to reveal the mechanism underlying the diverse effects of single mTORC1 and dual mTORC1/C2 inhibitors on the antigen presenting capacity of DCs.

Our findings describe for the first time, that mTOR kinase activity is increased upon RLR stimulation of human DCs. Furthermore, we show that mTOR plays an essential role in the regulation of RLR-mediated antiviral and pro-inflammatory cytokine production of human DCs. Here we also demonstrate that rapamycin greatly reduces the capacity of RLR-stimulated DCs to promote T cell responses, however the dual mTORC1/mTORC2 inhibitor failed to do so. Intriguingly, rapamycin and its derivative everolimus, which are commonly used for maintaining immunosuppression following solid organ transplantation has been proven to be effective in the management of viral infections that result from reactivation of latent viruses in transplant recipients (59). Very recently rapamycin (also known as sirolimus) has been also suggested as a potential candidate for treating COVID-19 infected patients based on a network-based drug repurposing model (60). However, further studies and clinical trials are needed to evaluate the effectiveness of mTOR inhibitors for protection against viral infections. Rapamycin is also used to induce the *ex vivo* generation of tolerogenic DCs, which are promising candidates for treatment of allergic diseases, for prevention of transplant rejection and are in clinical trials for therapy of autoimmune disorders (61). Meanwhile, dual mTOR kinase inhibitors are being tested in clinical trials to treat various types of human cancer, to prevent organ rejection and to control autoimmune diseases (62). These previous findings and our novel observations suggest that the distinct impacts of first and second generation mTOR inhibitors on immune cell functionality should be considered in the development of new generation mTOR inhibitors for any clinical applications.

## Data Availability Statement

All datasets presented in this study are included in the article/Supplementary Material.

## Ethics Statement

The collection of human heparinized leukocyte-enriched buffy coat samples complied with the guidelines of the Helsinki Declaration and was approved by National Blood Transfusion Service and the Regional and Institutional Ethics Committee of the University of Debrecen, Faculty of Medicine (OVSzK 3572-2/2015/5200, Hungary).

## Author Contributions

KP and TF designed the research, performed experiments, analyzed and interpreted data, and wrote the manuscript. BÁ, DB, and KB performed experiments and participated in data analysis. AS, TT, and ZV provided conceptual insight and revised the manuscript. KP and AB contributed with essential reagents. All authors reviewed and approved the manuscript.

## Conflict of Interest

The authors declare that the research was conducted in the absence of any commercial or financial relationships that could be construed as a potential conflict of interest.

## Abbreviations

BM-DC: bone marrow-derived DC
cDC: conventional DC
DC: dendritic cell
HIF1A: hypoxia-inducible factor 1-alpha
HK2: hexokinase 2
IFN: interferon
LDHA: lactate dehydrogenase A
MAPK: mitogen activated protein kinase
MDA5: melanoma differentiation-associated protein 5
moDC: monocyte-derived DC
mTOR(C): mammalian target of rapamycin (complex)
p70S6k: p70S6 kinase
pDC: plasmacytoid DC
polyI:C: polyinosinic:polycytidylic acid
PRR: pattern recognition receptor
RIG-I: retinoic-acid inducible gene I
RLR: RIG-I-like receptors
TBK1: tank-binding kinase 1
TLR: toll-like receptors
VSV: vesicular stomatitis virus.
