# Supplementary material for: Regulation of RLR-Mediated Antiviral Responses of Human Dendritic Cells by mTOR
Source: Front Immunol. 2020 Sep 11;11:572960. doi: 10.3389/fimmu.2020.572960 (PMC7516067; doi:10.3389/fimmu.2020.572960)
Supplement: Supplementary file 1 [file Table_1.docx]

Supplementary Material

Regulation of RLR-mediated antiviral responses of human dendritic cells by mTOR

Tünde Fekete, Beatrix Ágics, Dóra Bencze, Krisztián Bene, Antónia Szántó, Tünde Tarr, Zoltán Veréb, Attila Bácsi, Kitti Pázmándi*

* Correspondence: Kitti Pazmandi: pazmandikitti@yahoo.de

***Supplementary Figures***

**
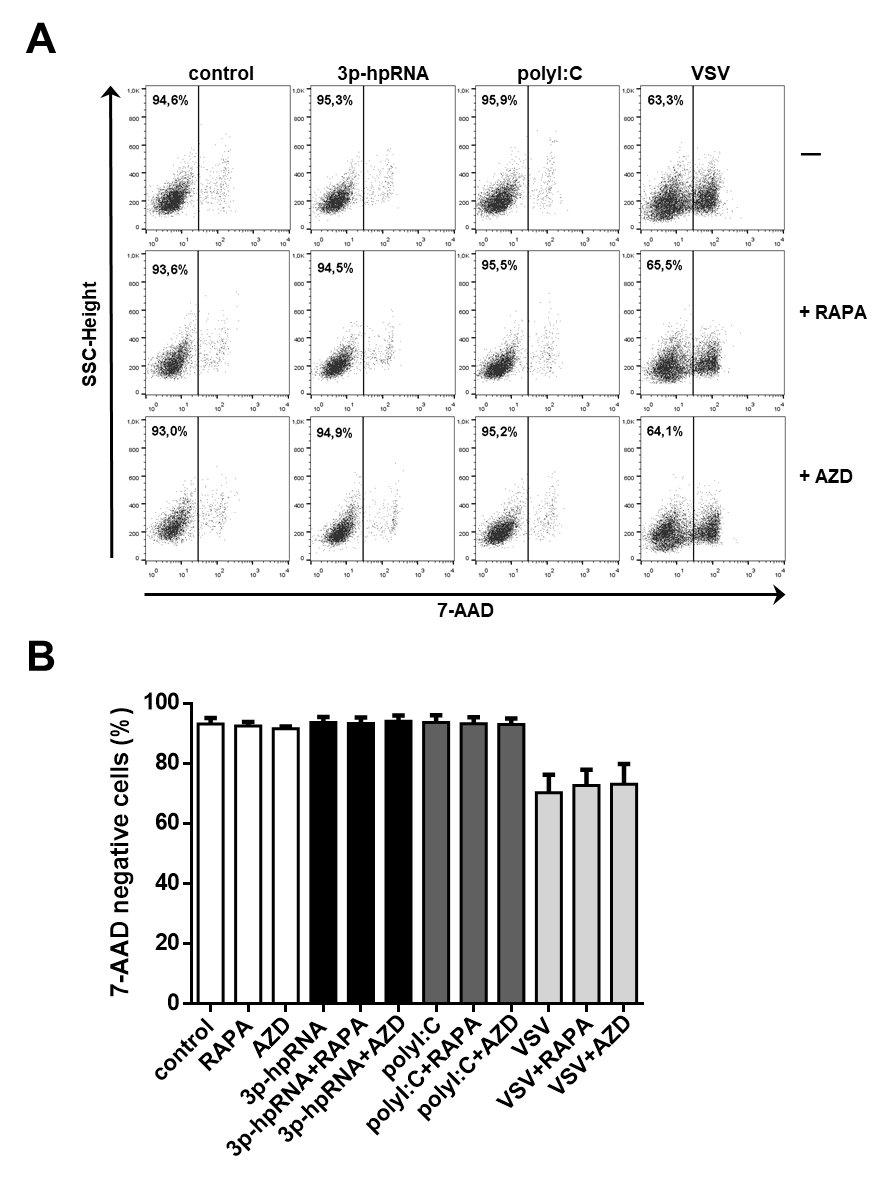
**

**Supplementary Figure 1.** Inhibition of mTOR functionality does not influence the viability of moDCs. Immature moDCs were pre-treated with vehicle control, 100 nM rapamycin (RAPA) or 100 nM AZD8055 (AZD) for 2 h and then stimulated with 3p-hpRNA (0.5 μg/ml), polyI:C (1 μg/ml) or VSV (MOI 1). After 24 hours cell viability was analyzed by flow cytometry. (A) Representative dot plots are shown, where numbers indicate the percentage of 7-aminoactinomycin D (7-AAD) negative viable cells. (B) Bar graphs show the mean ± SD of four independent experiments.


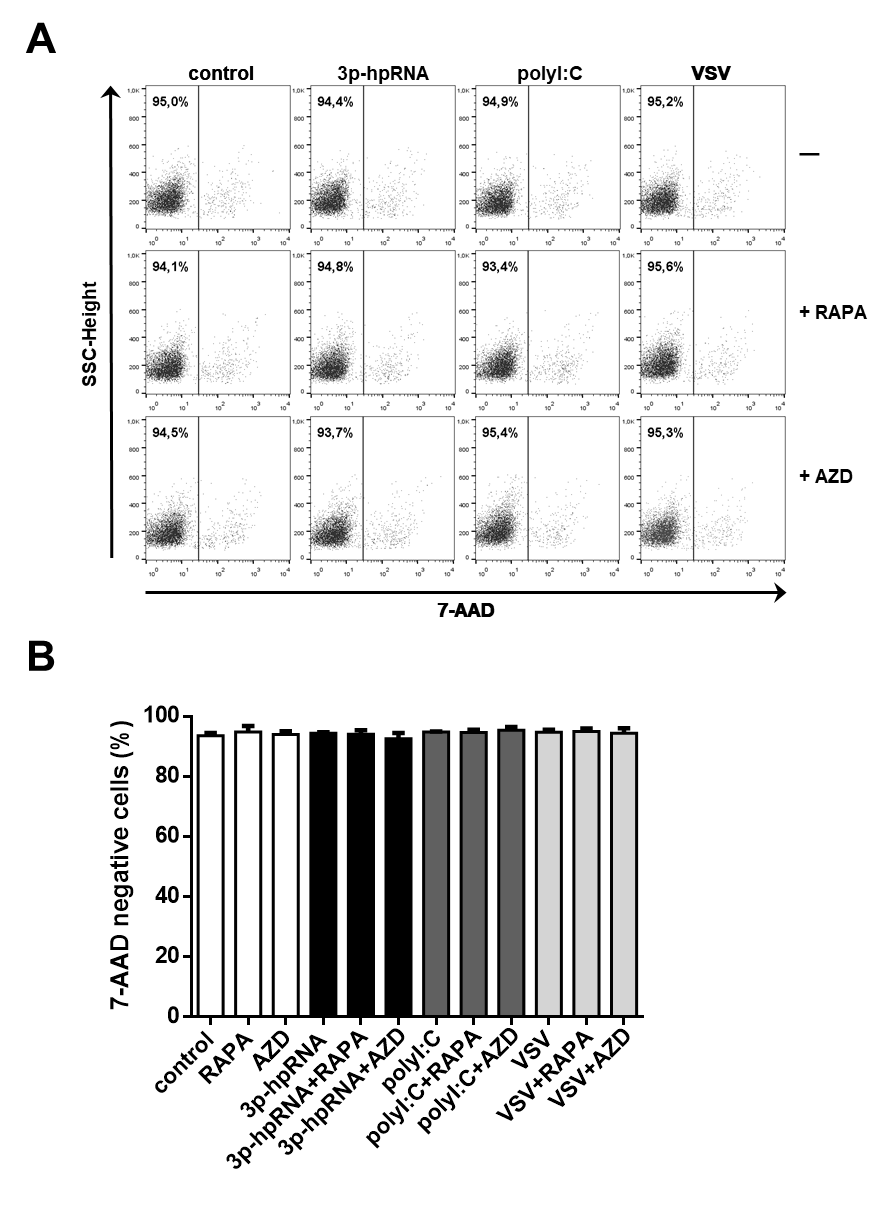


**Supplementary Figure 2.** Inhibition of mTOR functionality does not influence the viability of GEN2.2 cells. GEN2.2 cells were pre-treated with vehicle control, 100 nM rapamycin (RAPA) or 100 nM AZD8055 (AZD) for 2 h and then stimulated with 3p-hpRNA (0.5 μg/ml), polyI:C (1 μg/ml) or VSV (MOI 1). After 6 hours cell viability was analyzed by flow cytometry. (A) Representative dot plots are shown, where numbers indicate the percentage of 7-aminoactinomycin D (7-AAD) negative viable cells. (B) Bar graphs show the mean ± SD of four independent experiments.

***
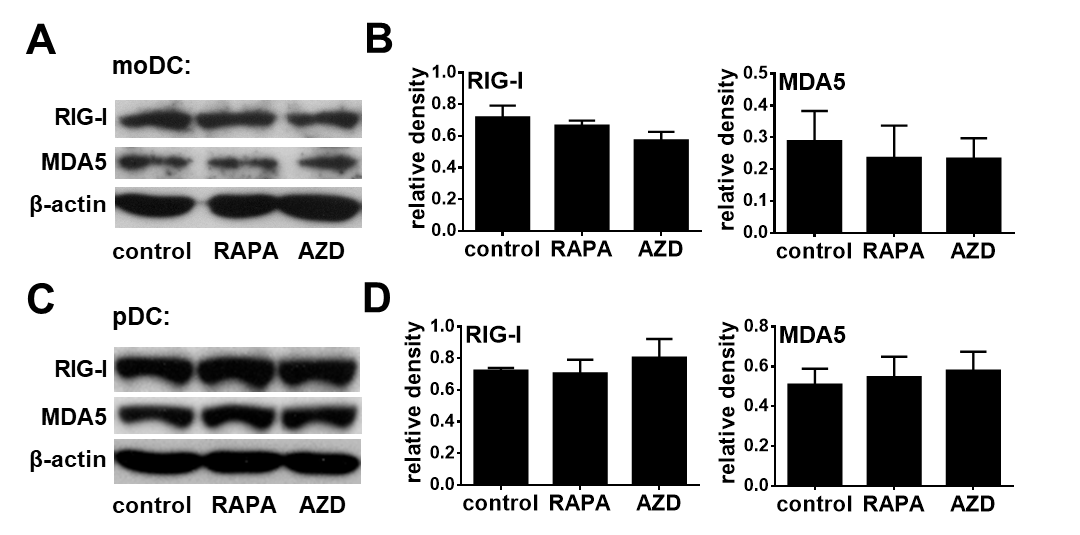
***

**Supplementary Figure 3.** The inhibition of mTOR does not influence the protein level of RLRs in moDCs and GEN2.2 cells. Immature moDCs (**A**,**B**) or GEN2.2 cells (**C**,**D**) were pre-conditioned with vehicle control, 100 nM rapamycin (RAPA) or 100 nM AZD8055 (AZD) for 2 h then protein levels of RIG-I, MDA5 and β-actin were assessed by western blot analysis. (**A**,**C**) Representative blots are shown. (**B**,**D**) Bar graphs show the relative densities normalized to β-actin and represent the mean ± SD of at least 3 independent experiments.

**
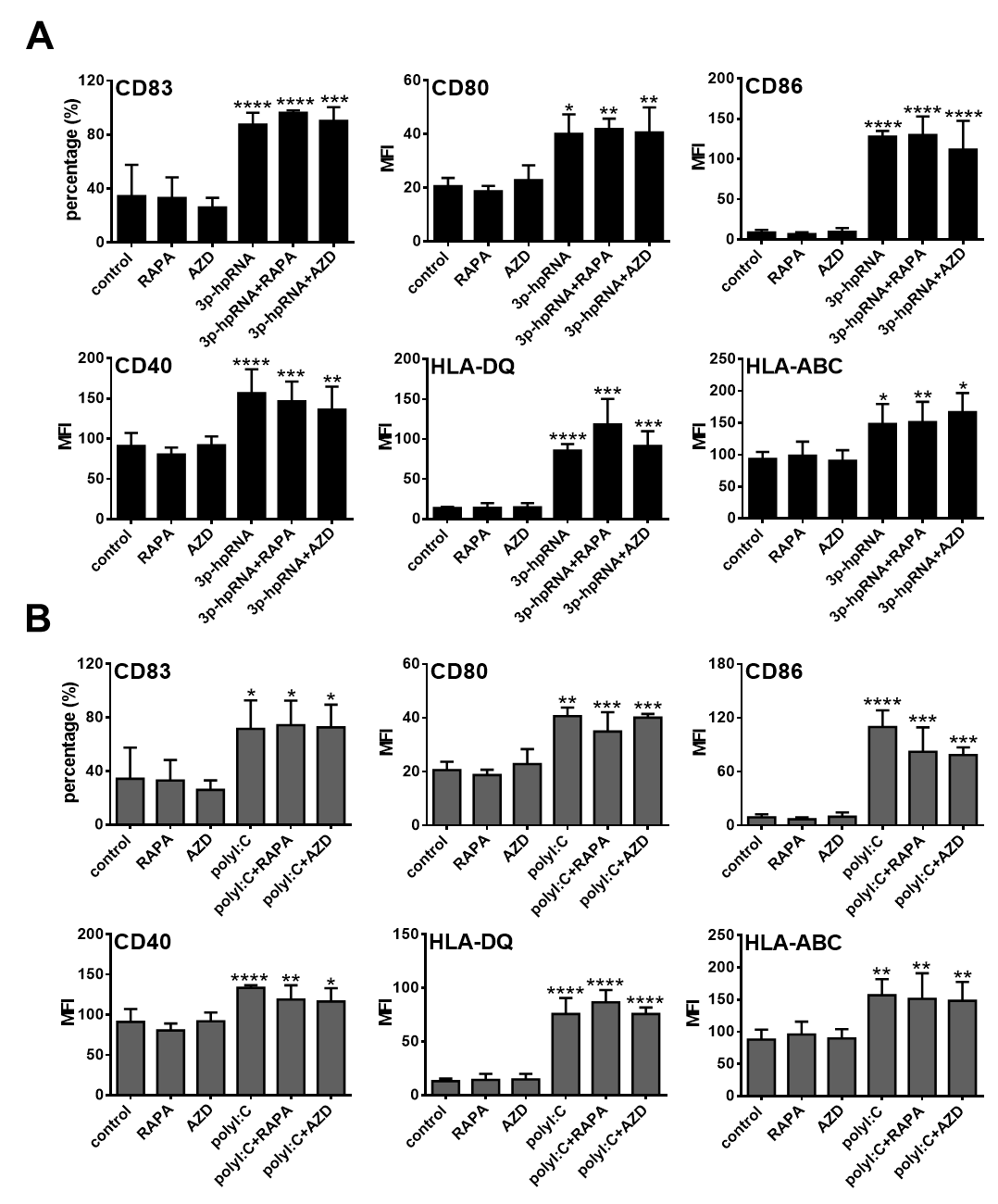
**

**Supplementary Figure 4.** The RLR-mediated expression of cell surface molecules is not affected by mTOR inhibition in moDCs. Immature moDCs were pre-treated with vehicle control, 100 nM rapamycin (RAPA) or 100 nM AZD8055 (AZD) for 2 h then stimulated with 3p-hpRNA (0.5 μg/ml) (A) or polyI:C (1 μg/ml) (B) for 24 h. The changes in the expression level of cell surface molecules were monitored by flow cytometry. Bar graphs represent the mean ± SD of at least 4 independent experiments. Data were analyzed using one-way ANOVA followed by Bonferroni’s post-hoc test. *p<0.05, **p<0.01, ***p<0.01, ****p<0.0001 vs. control.


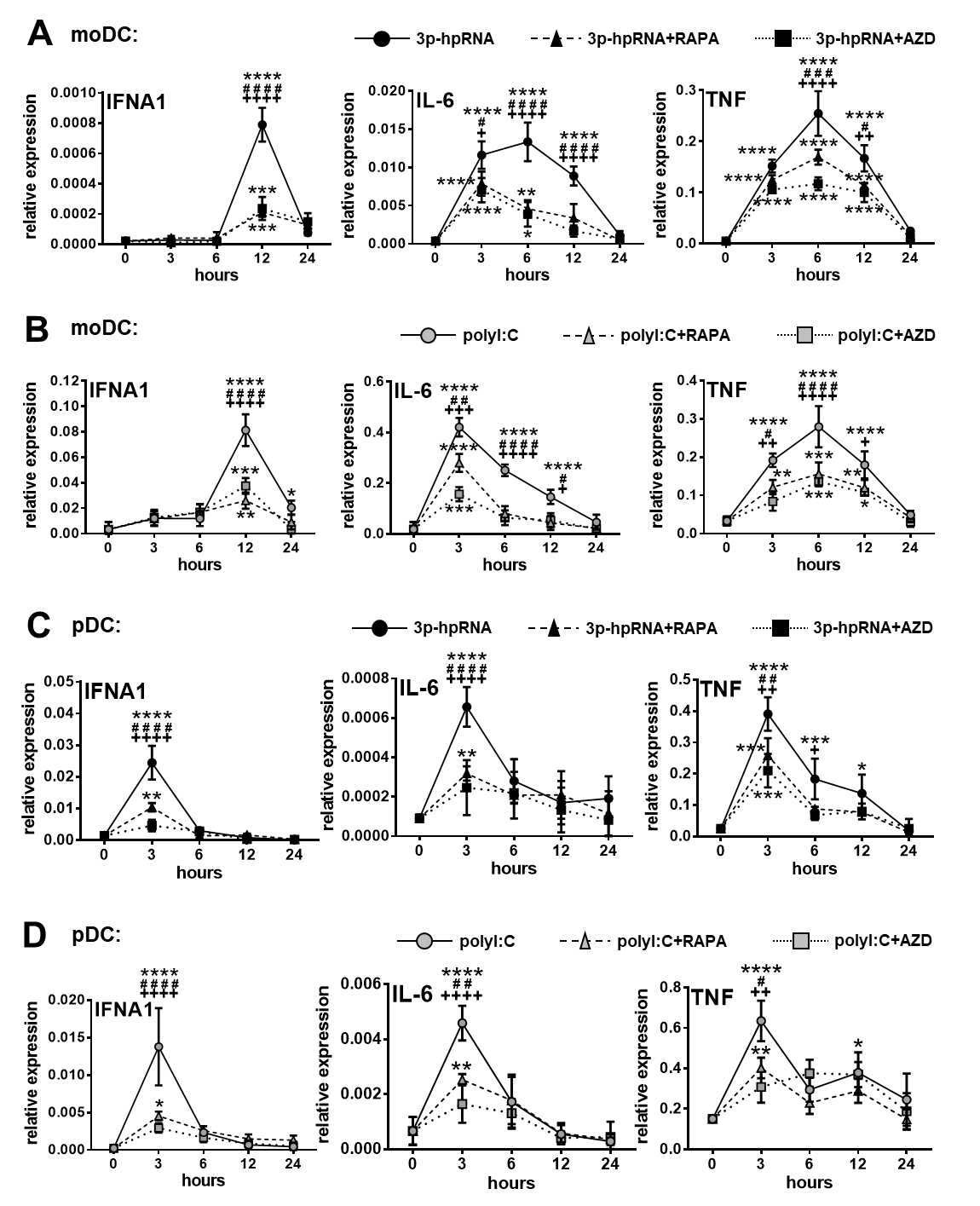


**Supplementary Figure 5**. The RLR-triggered upregulation of IFN-α and pro-inflammatory cytokine mRNA levels are decreased upon mTOR inhibition both in moDCs (**A**,**B**) and GEN2.2 (**C**,**D**) cells. Cells were pre-exposed to vehicle control, 100 nM rapamycin (RAPA) or 100 nM AZD8055 (AZD) for 2 h then stimulated with 3p-hpRNA (0.5 μg/ml) (**A**,**C**) or polyI:C (1 μg/ml) (**B**,**D**) in a time-dependent manner. The expression of *IFNA1*, *IL-6* and *TNF* was assessed at the mRNA level by real-time PCR. Time-course graphs represent the mean ± SD of at least 3 independent experiments. *p<0.05, **p<0.01, ***p<0.01 ****p<0.0001 vs. control; non-pre-treated vs. RAPA pre-treated: ^#^p<0.05, ^##^p<0.01, ^###^p<0.001, ^####^p<0.0001; non-pre-treated vs. AZD pre-treated: ^+^p<0.05, ^++^p<0.01, ^+++^p<0.001, ^++++^p<0.0001.


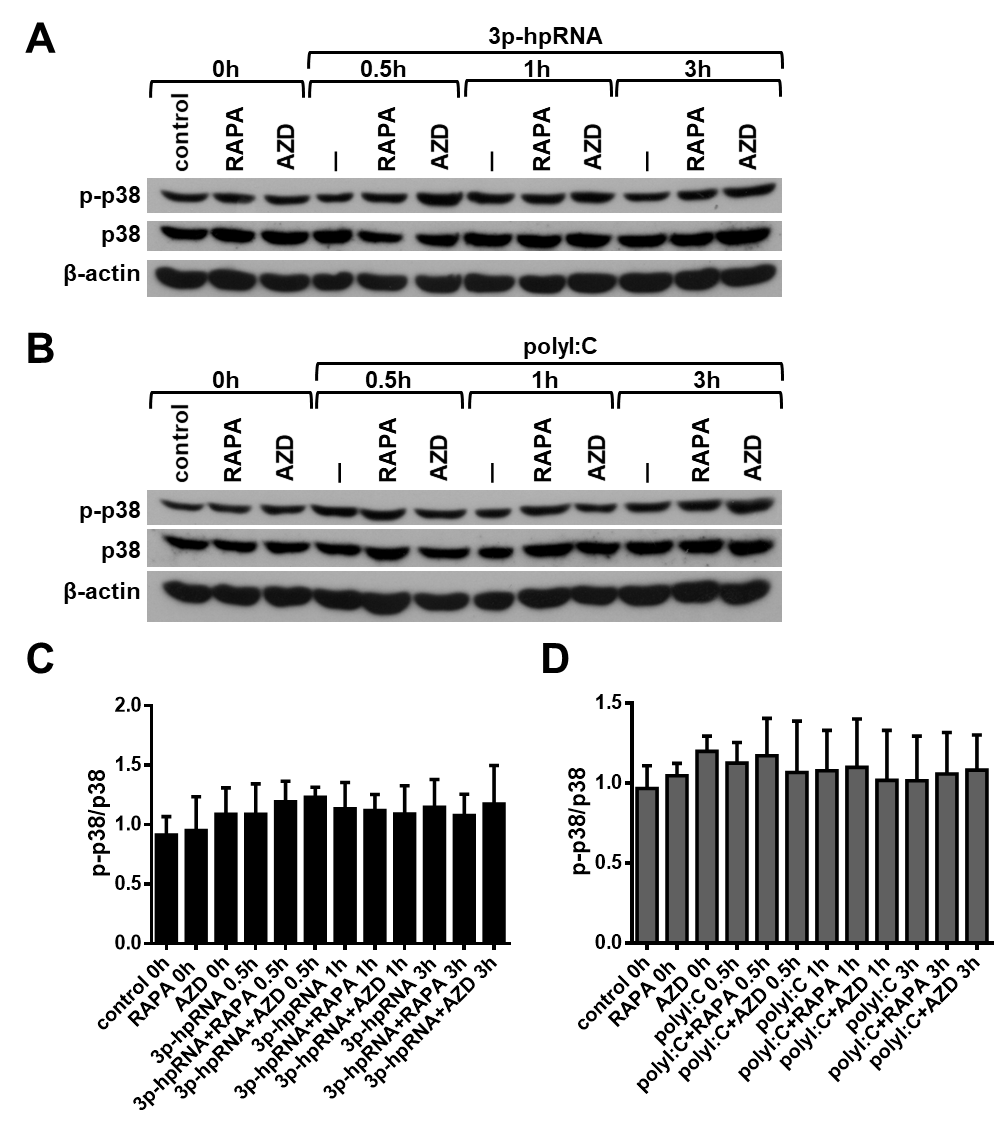


**Supplementary Figure 6.** The phosphorylation of p38 is not affected by RLR stimulation or mTOR inhibition in moDCs. Immature moDCs were pre-treated with vehicle control, 100 nM rapamycin (RAPA) or 100 nM AZD8055 (AZD) for 2 h then stimulated with 3p-hpRNA (0.5 μg/ml) (**A**,**C**) or polyI:C (1 μg/ml) (**B**,**D**) in a time-dependent manner. Kinetics of p38 phosphorylation were determined by western blotting. (**A**,**B**) Representative blots are shown. (**C**,**D**) Bar graphs represent the mean ± SD of at least 3 independent experiments.


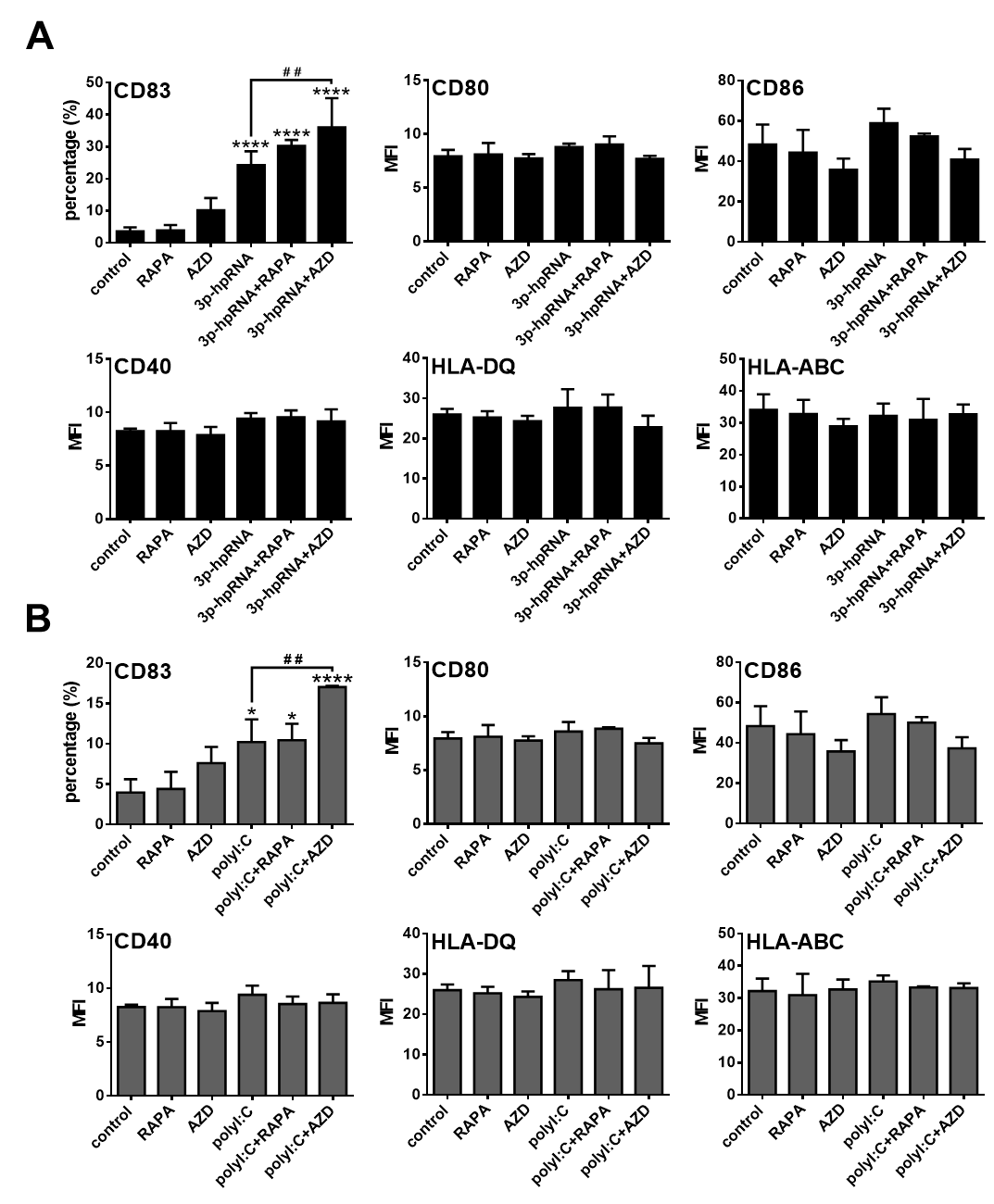


**Supplementary Figure 7**. The expression of cell surface molecules is only slightly and selectively modulated by mTOR inhibition in RLR-stimulated GEN2.2 cells. After pre-treatment with vehicle control, 100 nM rapamycin (RAPA) or 100 nM AZD8055 (AZD) for 2 h, GEN2.2 cells were stimulated with 3p-hpRNA (0.5 μg/ml) (A) or polyI:C (1 μg/ml) (B) for 24 h. The changes in the expression level of cell surface molecules were assessed by flow cytometry. Bar graphs represent the mean ± SD of at least 4 independent experiments. Data were analyzed using one-way ANOVA followed by Bonferroni’s post-hoc test. *p<0.05, ****p<0.0001 vs. control; ##p<0.01.


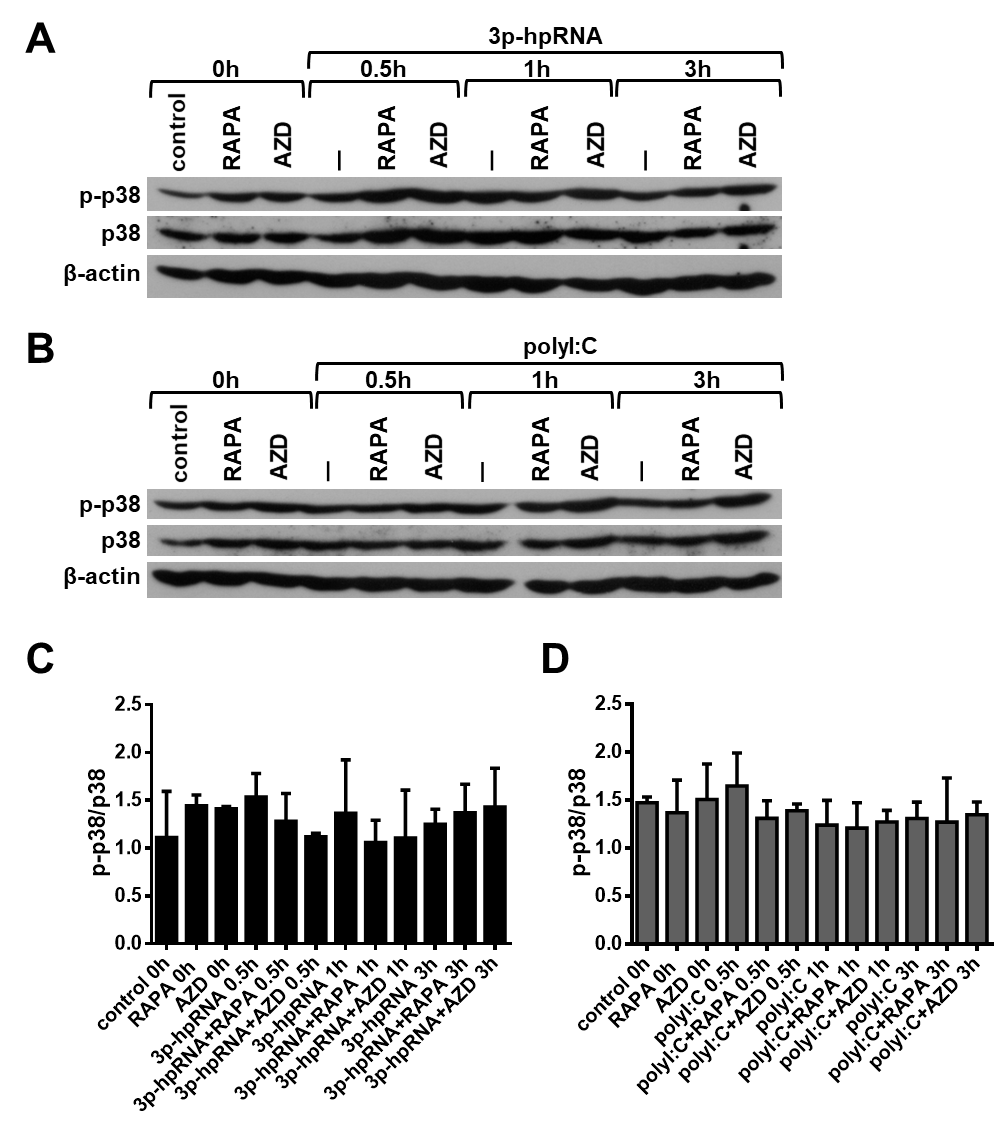


**Supplementary Figure 8.** The level of p38 phosphorylation did not change upon RLR stimulation or mTOR inhibition in GEN2.2 cells. Cells were pre-treated with vehicle control, 100 nM rapamycin (RAPA) or 100 nM AZD8055 (AZD) for 2 h then stimulated with 3p-hpRNA (0.5 μg/ml) (**A**,**C**) or polyI:C (1 μg/ml) (**B**,**D**) in a time-dependent manner. Kinetics of p38 phosphorylation were determined by western blotting. (**A**,**B**) Representative blots are shown. (**C**,**D**) Bar graphs represent the mean ± SD of at least 3 independent experiments.
